# Stability of a two-dimensional biomorphoelastic model for post-burn contraction

**DOI:** 10.1007/s00285-023-01893-w

**Published:** 2023-03-24

**Authors:** Ginger Egberts, Fred Vermolen, Paul van Zuijlen

**Affiliations:** 1 Delft Institute of Applied Mathematics, Delft University of Technology, Delft, The Netherlands; 2 Research Group Computational Mathematics (CMAT), Department of Mathematics and Statistics, University of Hasselt, Hasselt, Belgium; 3 Research Group Computational Mathematics (CMAT), University of Hasselt, Hasselt, Belgium; 4 Data Science Institute (DSI), University of Hasselt, Hasselt, Belgium; 5 Burn Centre and Department of Plastic, Reconstructive and Hand Surgery, Red Cross Hospital, Beverwijk, The Netherlands; 6 Department of Plastic, Reconstructive and Hand Surgery, Amsterdam UMC, location VUmc, Amsterdam Movement Sciences, Amsterdam, The Netherlands; 7 Pediatric Surgical Centre, Emma Children’s Hospital, Amsterdam UMC, location AMC and VUmc, Amsterdam, The Netherlands

**Keywords:** Burns, Wound contraction, Stability, Morphoelasticity, Moving-grid finite-element, 35B20, 35B35, 35G20, 35L65, 35M10, 35Q74, 35Q80, 35Q92, 35R37, 65C20, 65M12, 65M60, 65N12, 65N30, 74-10, 74L15, 92-10, 92C10, 92C17, 92C45, 93B18

## Abstract

We consider the stability analysis of a two-dimensional model for post-burn contraction. The model is based on morphoelasticity for permanent deformations and combined with a chemical-biological model that incorporates cellular densities, collagen density, and the concentration of chemoattractants. We formulate stability conditions depending on the decay rate of signaling molecules for both the continuous partial differential equations-based problem and the (semi-)discrete representation. We analyze the difference and convergence between the resulting spatial eigenvalues from the continuous and semi-discrete problems.

## Introduction

In recent decades, healthcare has made significant progress so that today patients can survive even severe burns. Nevertheless, burns are still life-threatening and difficult to assess and treat (Lang et al. 2019). These injuries will still significantly impact the quality of human life. Additional factors after a burn can include shock, infection, and prolonged stress and are of a physical, mental and social nature. Besides slow wound healing, the prevention of hypertrophic scars and contractures, which always cause a reduction in patient mobility, are significant challenges in burn treatment (Wang et al. 2018).

Almost all full-thickness burns lead to scarring. Deep burns leave no vital skin tissue, including the collagen network, cells (fibroblast), and vasculature. If the resulting scars entail contraction, these scars are subject to change in dimensions. A critical physiological aspect is the occurrence of burn contractures, which are contractions that cause reduced mobility of joints. Mostly, those scars require reconstructive surgery.

Burn healing comprises three partly overlapping phases: inflammation (reactive), proliferation (reparative), and maturation (remodeling). First, immune cells clear contaminants and pathogens within several hours after injury. Secreted growth factors stimulate cells to migrate to the wound from the intact peripheral dermis and subcutaneous tissue to the wound. This migration is a hallmark of proliferation; the cells multiply in the injured area and replace the fibrin network by regenerating collagen. During this phase, cells form a temporary spongy extracellular matrix (ECM) (granulation tissue) which is replaced by a solid matrix at a much later stage (remodeling). Collagen type III fills the granulation tissue as a provisional matrix early, and collagen type III is replaced by the embryonic collagen type I during remodeling. In this final stage, which can last for years, the scar matures and forms a balanced structure.

Post-burn contraction is one sub-process during the proliferative phase. Under the influence of growth factors, fibroblasts can differentiate into myofibroblasts (Desmoulière et al. 1993). Myofibroblasts produce a large amount of collagen to which the cells attach and pull forces. Furthermore, like fibroblasts, these cells stimulate both the production of the components of the new collagen-rich ECM and the release of matrix metalloproteinases (MMPs). Usually, myofibroblasts disappear by apoptosis when the wound closes (Desmoulière et al. 1995). However, if myofibroblasts persist in a closed wound, they continue to pull, produce excess collagen, and develop a hypertrophic scar (Tomasek et al. 2002). Thus, the biomechanical interaction of fibroblasts, myofibroblasts, growth factors, and collagen is essential in post-burn contraction and hypertrophy.

Various scientific disciplines study the prevention of contractures, including biology, the medical sciences, and mathematics. For a couple of decades, mathematical models have simulated wound healing. These models predict the behavior of experimental and clinical wounds and contractions (Tranquillo and Murray 1992; Olsen et al. 1995; Barocas and Tranquillo 1997; Dallon et al. 1999; McDougall et al. 2006; Koppenol 2017; Menon et al. 2017). We can divide most of these models into continuum hypothesis-based, discrete cell-based, and hybrid models (Koppenol 2017). Here, the discrete cell-based model type considers individual cell interactions compared to the continuum models considering cellular densities in a tissue. Hybrid modeling is classically defined as coupling a continuous approach with a discrete one to model a complex phenomenon that cannot be described in a standard homogeneous way mainly because of its inherent multiscale nature (Stéphanou and Volpert 2015). One subcategory of the continuum (partial differential equations-based) hypothesis-based models is the mechano-(bio)chemical model. This formalism and the hybrid model provided the basis for the biomorphoelastic model we use in this study, which Koppenol and Vermolen developed (Koppenol and Vermolen 2017). This biomorphoelastic model can simulate permanent post-burn contraction and reproduce the trends observed in real-life data.

Following up on our previous study (Egberts et al. 2021b), we analyze the stability of the biomorphoelastic model for post-burn contraction around equilibria in a two-dimensional setting. The model’s equations are nonlinear and multivariable, making multiple steady states possible. Some steady states might be unstable for specific parameter values, and the model might reach other steady states for linearly unstable parameter values. Therefore, this study aims to avoid the parametric dependence of stable and unstable solutions and to assess the local behavior around the steady states. We analyze the system of equations and provide stability conditions using Gershgorin’s theorem, where we cannot compute the eigenvalues exactly. As in Egberts et al. (2021b), we use a linear stability analysis with Fourier series, where the transformations represent perturbations around equilibria.

Further, we give the eigenvalues for a specific case. Biologically this means that the equilibria of the effective strain are determined. We distinguish between the continuous problem, which represents the solution to the system of partial differential equations, and the semi-discrete problem, which represents the solution of a semi-discrete solution method. In the latter case, the spatial finite difference method is carried out, whereas the time remains continuous. We show that the continuous system’s stability implies the semi-discrete system’s stability. Besides stability conditions, we pay attention to the effects of system instability on actual post-burn contraction. We also discuss specific parts of the model that can be adjusted to reproduce additional biological observations and the application of machine learning to make the application of the model for personalized healthcare possible. The results reported in this paper complement the stability analysis of the model’s one-dimensional counterpart.

The structure of this paper is as follows. Section 2 presents the mathematical model, and Sect. 3 presents the stability analysis. Then, Sect. 4 presents the validation of (in)stability. Finally, Sect. 5 presents the discussion and conclusion.

## Mathematical model

We consider the post-burn contraction mathematical model based on morphoelasticity (Koppenol and Vermolen 2017). Morphoelasticity is based on decomposing the total deformation gradient into a deformation gradient by growth or shrinkage, and deformation by mechanical forces (Hall 2008). These processes may occur simultaneously or consecutively. The advantage of this model is that it can simulate permanent deformation resulting from the contraction process in burn wound healing. The most important variable in this model is the skin displacement (${\textbf{u}}$), i.e., the variable that allows us to determine the area of the wound and, in later stages, the scar. This variable is estimated using a set of other variables that we can divide into biochemical and mechanical. The biochemical variables are the fibroblast density (*N*), the myofibroblast density (*M*), the signaling molecules concentration (*c*), and the collagen density ($\rho$), and for the mechanics, we have the displacement velocity (${\varvec{v}}$) and the effective strain ($\varvec{\varepsilon }$). These variables are each modeled by a partial differential equation (PDE). Hence, we consider continuous distributions and concentrations in contrast to agent-based modeling considering individual cells. In this section, we show and explain the equations that involve the material time derivative $\frac{\textrm{D}(\cdot )}{\textrm{D}t}$ and passive convection $z(\nabla \cdot {\varvec{v}})$, $z\in \{N,M,c,\rho ,{\varvec{v}}\}$. These concepts are introduced because the domain of computation is subject to displacement.

After an injury, the cascade of processes that characterize wound healing is initiated by releasing growth factors and cytokines (by immune cells) that we consider a collective of signaling molecules. These signaling molecules influence cell proliferation (as an activator-inhibitor), myofibroblast differentiation, chemotaxis, and the synthesis and decay of collagen. For the cells, we consider migration towards the gradient of the signaling molecules (Postlethwaite et al. 1987) by a minimal model for chemotaxis (Hillen and Painter 2008), and cell density-dependent Fickian diffusion for the inclusion of random walk. The equations for the cell densities of fibroblasts and myofibroblasts also contain a logistic-like cell proliferation term. The equations are given by:

1 $$\begin{aligned}{} & {} \frac{\textrm{D}N}{\textrm{D} t} + N(\nabla \cdot {\varvec{v}}) = -\nabla \cdot \left( - D_F (N+M) \nabla N + \chi _F N \nabla c\right) \nonumber \\{} & {} + r_F \left[ 1+\frac{r_F^{\text {max}}c}{a_c^{I}+c} \right] [1-\kappa _F (N+M)] N^{1+q} - k_F c N - \delta _N N, \end{aligned}$$

2 $$\begin{aligned}{} & {} \frac{\textrm{D}M}{\textrm{D} t} + M(\nabla \cdot {\varvec{v}}) = -\nabla \cdot \left( - D_F (N + M)\nabla M + \chi _F M \nabla c\right) \nonumber \\{} & {} + r_F \left[ \frac{[1 + r_F^{\text {max}}]c}{a_c^{I} + c} \right] [1 - \kappa _F (N+M)] M^{1+q} + k_F c N - \delta _M M. \end{aligned}$$

Here, $D_F$ represents the (myo)fibroblast diffusion coefficient, which accounts for a random walk, and $\chi _F$ is the chemotactic parameter, $r_F$ is the cell division rate, $r_F^\text {max}$ is the maximum factor of cell division rate enhancement because of the presence of the signaling molecules, $a_c^I$ is the concentration of the signaling molecules that cause half-maximum enhancement of the cell division rate, $\kappa _F(N+M)$ represents the reduction in the cell division rate because of crowding (Vande Berg et al. 1989), *q* is a fixed exponent, $k_F$ is the signaling molecule-dependent cell differentiation rate constant of fibroblasts into myofibroblasts, and $\delta _N$, $\delta _M$ represent the apoptosis rates of the fibroblasts and myofibroblasts, respectively. An essential difference between these equations is that myofibroblasts are assumed to only proliferate in the presence of signaling molecules.

The following equations describe the evolution of the signaling molecules and collagen. The signaling molecules only migrate because of (Fickian) diffusion, and collagen is not subject to active migration. In both equations, (myo) fibroblasts are responsible for the secretion, and MMPs are responsible for the breakdown:

3 $$\begin{aligned}{} & {} \frac{\textrm{D}c}{\textrm{D} t} + c(\nabla \cdot {\varvec{v}}) = \nabla \cdot (D_c \nabla c) + k_c \left[ \frac{c}{a_c^{II} + c} \right] [N + \eta ^I M] - \delta _c \frac{[N + \eta ^{II}M]\rho }{1 + a_c^{III}c} c, \end{aligned}$$

4 $$\begin{aligned}{} & {} \frac{\textrm{D}\rho }{\textrm{D} t} + \rho (\nabla \cdot {\varvec{v}}) = k_\rho \left[ 1 + \left[ \frac{k_\rho ^{\text {max}}c}{a_c^{IV} + c} \right] \right] [N + \eta ^I M] -\delta _\rho \frac{[N + \eta ^{II}M]\rho }{1 + a_c^{III}c} \rho . \end{aligned}$$

Here $D_c$ is the Fickian diffusion coefficient of the signaling molecules, $k_c$ is the maximum net secretion rate of the signaling molecules, $\eta ^I$ is the ratio of myofibroblasts to fibroblasts in the maximum secretion rate of the signaling molecules and collagen, $a_c^{II}$ is the concentration of the signaling molecules that causes the half-maximum net secretion rate of the signaling molecules, $\delta _c$ is the proteolytic breakdown rate parameter of the signaling molecules, $\eta ^{II}$ is the ratio of myofibroblasts to fibroblasts in the secretion rate of the MMPs and $1\,+\,a_c^{III}c$ represents the inhibition of the secretion of the MMPs. Further, $k_\rho$ is the collagen secretion rate, $k_\rho ^{\text {max}}$ is the maximum factor of secretion rate enhancement because of the presence of the signaling molecules, $a_c^{IV}$ is the concentration of the signaling molecules that cause the half-maximum enhancement of the secretion rate of collagen and $\delta _\rho$ is the degradation rate of collagen. A generic MMP affects the reaction kinetics of the signaling molecules and collagen, and we assume it is always at a local equilibrium concentration. This assumption avoids even more complexity and additional unknown and undocumented parameter values.

We capture the mechanics of the model by two PDEs for the displacement velocity (${\varvec{v}}$) and the effective strain ($\varvec{\varepsilon }$). In the equation for displacement velocity, the Cauchy stress tensor $\sigma$ relates to the effective strain and displacement velocity gradients by a visco-elastic constitutive relation. The body force ${\textbf{f}}$ is generated by a pulling force on the ECM by myofibroblasts, which we assume to be proportional to the product of the cell density of the myofibroblasts and a function of the concentration of collagen^1^:

5 $$\begin{aligned} \rho _t \left( \frac{\text {D} {\varvec{v}}}{\text {D} t} + {\varvec{v}}(\nabla \cdot {\varvec{v}}) \right) - \nabla \cdot \sigma = {\textbf{f}} = \nabla \cdot \left( \frac{\xi M\rho }{R^2 + \rho ^2}{\textbf{I}} \right) . \end{aligned}$$

This equation represents the balance of momentum. Here $\rho _t$ represents the total mass density of the dermal tissues, $\xi$ is the generated stress per unit cell density and the inverse of the unit collagen concentration, and *R* is a constant. Despite many studies neglecting inertial effects, we have kept the inertia terms (the time derivative) closer to the underlying physics. From a mechanical point of view, we assume the tissue to be isotropic and homogeneous, except for a dependency of the stiffness on the local collagen density (Ramtani 2004). The visco-elastic relation for the Cauchy stress tensor is:

6 $$\begin{aligned} \sigma = \mu _1\text {sym}(\nabla {\varvec{v}}) + \mu _2(\text {tr}(\text {sym}(\nabla {\varvec{v}})){\textbf{I}}) + \frac{E\sqrt{\rho }}{1 + \nu }\left[ \varvec{\varepsilon } + \text {tr}(\varvec{\varepsilon })\frac{\nu }{1 - 2\nu }{\textbf{I}}\right] , \end{aligned}$$

where $\mu _1,\mu _2$ are the shear and bulk viscosity, respectively, $E\sqrt{\rho }$ represents Young’s modulus (stiffness), and $\nu$ is the Poisson’s ratio. Despite possibly obtaining large deformations in the tissue, we use linear elasticity to avoid the need to include additional input parameters.

We incorporate permanent deformation because of microstructural changes in the tissue via morphoelasticity. This tensor-based approach is also commonly used in the growth of tissues (such as tumors). For the constitutive law, we use Hooke’s law which assumes a linear relationship between stress and strain. Following (Koppenol and Vermolen 2017), we assume the effective Eulerian strain to be modelled by

7 $$\begin{aligned} \frac{\text {D}\varvec{\varepsilon }}{\text {D}t} + \varvec{\varepsilon }\,\text {skw}(\nabla {\varvec{v}}) - \text {skw}(\nabla {\varvec{v}})\varvec{\varepsilon } + (\text {tr}(\varvec{\varepsilon }) - 1)\text {sym}(\nabla {\varvec{v}}) = -\zeta \frac{[N + \eta ^{II}M]c}{1 + a_c^{III}c}\varvec{\varepsilon }. \end{aligned}$$

Here, $\zeta$ is the rate of morphoelastic change (i.e., the rate at which the effective strain changes actively over time). In particular, the tensor for contraction depends on the product of the concentration of the MMPs, the concentration of the signaling molecules, and it is inversely proportional to the collagen density (note that the collagen density drops out because of the linear dependence of the equilibrium MMP concentration on the collagen density). Further, the linear strain evolution equation (7) provides an appropriate description provided that all components of $\varvec{\varepsilon }$ are small. Therefore, as well as eqn. (6), if $\varvec{\varepsilon }\rightarrow {\mathcal {O}}(1)$, then the model requires another constitutive law.

### Further assumptions and boundary conditions

For the two-dimensional setting, we locate the *xy*-plane parallel to the surface of the skin and

8 $$\begin{aligned} {\varvec{v}} = \begin{bmatrix} v_1\\ v_2 \end{bmatrix}, \qquad \text {and} \qquad \varvec{\varepsilon } = \begin{bmatrix} \varepsilon _{11} &{} \varepsilon _{12}\\ \varepsilon _{21} &{} \varepsilon _{22} \end{bmatrix}. \end{aligned}$$

Variations over skin depth are disregarded; hence the computations are done on an arbitrary skin depth.

The domain, and the initial conditions, are symmetrical; hence the solution inherits this property. Therefore, we can perform calculations on a reduced domain to benefit from a computational workload.

We define the computational domain’s boundary by ${\overline{\Omega }}_{{\textbf{x}},t} = \Gamma _{{\textbf{x}},t}^o \bigcup \Gamma _{{\textbf{x}},t}^h \bigcup \Gamma _{{\textbf{x}},t}^v$. Here $\Gamma ^o$ represents the outer nonsymmetrical boundaries, $\Gamma ^h$ represents the horizontal symmetrical boundary where $y = 0$, and $\Gamma ^v$ represents the vertical symmetrical boundary where $x = 0$. For the constituents of the dermal layer, the following boundary conditions hold for all time *t* and for all

9 $$\begin{aligned} {\textbf{x}} \in \Gamma _{{\textbf{x}},t}^o&: \quad N({\textbf{x}},t) = {\overline{N}}, \quad M({\textbf{x}},t) = {\overline{M}}, \quad \text {and} \quad c({\textbf{x}},t) = {\overline{c}}, \end{aligned}$$

10 $$\begin{aligned} {\textbf{x}} \in \Gamma _{{\textbf{x}},t}^p&: \quad {\textbf{J}}_{N/M/c} \cdot {\textbf{n}} = 0, \end{aligned}$$

where $p\in \{h,v\}$ and ${\textbf{n}}$ is the outward pointing normal vector. We use similar conditions for the mechanical part of the model, that is, for all time *t* and for all

11 $$\begin{aligned} {\textbf{x}} \in \Gamma _{{\textbf{x}},t}^o&: \quad {\varvec{v}}({\textbf{x}},t) = 0, \end{aligned}$$

12 $$\begin{aligned} {\textbf{x}} \in \Gamma _{{\textbf{x}},t}^p&: \quad {\varvec{v}}\cdot {\textbf{n}} = 0 \quad \text {and} \quad (\sigma \cdot {\textbf{n}})\cdot \tau = 0, \end{aligned}$$

where $\tau$ is the tangential vector. It is not allowed to specify boundary conditions for $\rho$ and $\varvec{\varepsilon }$ because of overdetermination since the equations for $\rho$ and $\varvec{\varepsilon }$ are ordinary differential equations with respect to time *t*. We vary the initial conditions around ${\overline{N}}, 0, 0, {\overline{\rho }}, 0$ for $N({\textbf{x}},0), M({\textbf{x}},0), c({\textbf{x}},0), \rho ({\textbf{x}},0)$, and $v_1({\textbf{x}},0)$, respectively, and we set $v_2({\textbf{x}},0) = -\,v_1({\textbf{x}},0)$ to ensure symmetry. Further, we use $\varvec{\varepsilon }({\textbf{x}},0) = 0$.

## Linear stability of the model

We analyze the stability of the two-dimensional biomorphoelastic model for post-burn contraction to understand the a priori behavior of the solution. We cannot derive the (exact) solution to coupled nonlinear partial differential equations system. In line with our previous stability analysis in ${\mathbb {R}}^1$, we first analyze the linear stability of the continuous problem. Further, we also analyze the stability of the numerical approximation. We consider the following linearised equations around equilibria $(N, M, c, \rho , v_1, v_2, \varepsilon _{11}, \varepsilon _{12}, \varepsilon _{22}) = ({\overline{N}}, 0, 0, {\overline{\rho }}, 0, 0, \overline{\varepsilon _{11}}, \overline{\varepsilon _{12}}, \overline{\varepsilon _{22}})$, where ${\overline{N}}, {\overline{\rho }} \in {\mathbb {R}}_{\ge 0}$ and $\overline{\varepsilon _{11}}, \overline{\varepsilon _{12}}, \overline{\varepsilon _{22}} \in {\mathbb {R}}$:

13 $$\begin{aligned}{} & {} \frac{\partial {\hat{N}}}{\partial t} + {\overline{N}} \nabla \cdot \left[ -D_F \nabla {\hat{N}} + \chi _F \nabla {\hat{c}} \right] -r_F {\overline{N}}^q \left[ (1 + q)(1 - \kappa _F {\overline{N}}) - \kappa _F {\overline{N}} \right] {\hat{N}} \, \nonumber \\{} & {} \quad +\delta _N {\hat{N}} \,+ r_F \kappa _F {\overline{N}}^{1 + q} {\hat{M}} - {\overline{N}} \left[ \frac{r_F r^{\max }}{a_c^{I}}(1 - \kappa _F {\overline{N}}) {\overline{N}}^q - k_F \right] {\hat{c}} = 0,\nonumber \\{} & {} \quad \frac{\partial {\hat{M}}}{\partial t} - D_F {\overline{N}} \nabla \cdot (\nabla {\hat{M}}) + \delta _M {\hat{M}} - k_F {\overline{N}} {\hat{c}} = 0,\nonumber \\{} & {} \quad \frac{\partial {\hat{c}}}{\partial t} - D_c \nabla \cdot (\nabla {\hat{c}}) + {\overline{N}} \left[ \delta _c {\overline{\rho }} - \frac{k_c}{a_c^{II}} \right] {\hat{c}} = 0,\nonumber \\{} & {} \quad \frac{\partial {\hat{\rho }}}{\partial t} +\delta _\rho {\overline{\rho }}^2 (\eta ^{II} - \eta ^I) {\hat{M}} - \delta _\rho {\overline{\rho }}^2 {\overline{N}} \left[ \frac{k_\rho ^{max}}{a_c^{IV}} + a_c^{III} \right] {\hat{c}} + 2 \delta _\rho {\overline{N}} {\overline{\rho }} {\hat{\rho }} = 0, \end{aligned}$$

for the chemical part of the model, where we used that $k_\rho = \delta _\rho {\overline{\rho }}^2$ must hold in equilibrium, and

14 $$\begin{aligned} \begin{aligned} \rho _t \frac{\partial {\hat{v}}_1}{\partial t} - \left( \mu _1 + \mu _2\right) \frac{\partial ^2 {\hat{v}}_1}{\partial x^2} - \frac{\mu _1}{2} \frac{\partial ^2 {\hat{v}}_1}{\partial y^2} - \left[ \frac{\mu _1}{2} + \mu _2 \right] \frac{\partial ^2 {\hat{v}}_2}{\partial x \partial y} \, \ \frac{E \sqrt{{\overline{\rho }}}}{1 + \nu } \left[ \frac{\partial {\hat{\varepsilon }}_{12}}{\partial y} + \frac{\partial {\hat{\varepsilon }}_{11}}{\partial x} + \frac{\nu }{1 - 2\nu }\left[ \frac{\partial {\hat{\varepsilon }}_{11}}{\partial x} + \frac{\partial {\hat{\varepsilon }}_{22}}{\partial x} \right] \right] \ \frac{E}{2 \sqrt{{\overline{\rho }}}(1 + \nu )} \left[ \overline{\varepsilon _{12}} + \overline{\varepsilon _{11}} + \frac{\nu }{1 - 2\nu }(\overline{\varepsilon _{11}} + \overline{\varepsilon _{22}})\right] \frac{\partial {\hat{\rho }}}{\partial x} - \xi \frac{{\overline{\rho }}}{R^2 + {\overline{\rho }}^2} \frac{\partial {\hat{M}}}{\partial x} = 0, \end{aligned}\qquad \nonumber \\ \end{aligned}$$

for $v_1$ (the equation for $v_2$ is similar, where *x*, *y* and ${\hat{v}}_1, {\hat{v}}_2$ are interchanged), and

15 $$\begin{aligned} \begin{aligned} \frac{\partial {\hat{\varepsilon }}_{11}}{\partial t} + \overline{\varepsilon _{12}} \left[ \frac{\partial {\hat{v}}_2}{\partial x} - \frac{\partial {\hat{v}}_1}{\partial y} \right] + (\overline{\varepsilon _{11}} + \overline{\varepsilon _{22}} - 1) \frac{\partial {\hat{v}}_1}{\partial x} + \zeta {\overline{N}} \overline{\epsilon _{11}} {\hat{c}} = 0,\\ \frac{\partial {\hat{\epsilon }}_{12}}{\partial t} + \overline{\epsilon _{22}} \frac{\partial {\hat{v}}_2}{\partial x} + \overline{\epsilon _{11}} \frac{\partial {\hat{v}}_1}{\partial y} - \frac{1}{2} \left[ \frac{\partial {\hat{v}}_2}{\partial x} + \frac{\partial {\hat{v}}_1}{\partial y}\right] + \zeta {\overline{N}} \overline{\epsilon _{12}} {\hat{c}} = 0, \end{aligned} \end{aligned}$$

for the effective strains ${\hat{\varepsilon }}_{11}, {\hat{\varepsilon }}_{12}$ (the equation for ${\hat{\varepsilon }}_{22}$ is similar as for ${\hat{\varepsilon }}_{11}$, where *x*, *y* and ${\hat{v}}_1, {\hat{v}}_2$ are interchanged). In Eqs. (13)–(15), ${\hat{N}}, {\hat{M}}, {\hat{c}}, {\hat{\rho }}, {\hat{v}}_1, {\hat{v}}_2$, ${\hat{\varepsilon }}_{11}$, ${\hat{\varepsilon }}_{12}$, and ${\hat{\varepsilon }}_{22}$ are variations around the equilibria. Hence, $N({\textbf{x}},t) = {\overline{N}} + {\hat{N}}({\textbf{x}},t)$, etc.

Note that we only consider the equation for $\varepsilon _{12}$ and not $\varepsilon _{21}$. We demonstrate that if the strain tensor $\varvec{\varepsilon }$ is initially symmetric, then it remains symmetric at all later times (Egberts et al. 2021a).

### Theorem 1

Let Eq. (7) hold on an open Lipschitz domain $\Omega$ for $t>0$. Suppose that $\varvec{\varepsilon }$ is symmetric on $t=0$, then $\varvec{\varepsilon }$ remains symmetric for $t>0$.

### Proof

Taking the transpose of Eq. (7), gives

16 $$\begin{aligned} \begin{aligned} \frac{\text {D}\varvec{\varepsilon }}{\text {D}t} + \varvec{\varepsilon } \, \text {skw}(\nabla {\varvec{v}}) - \text {skw}(\nabla {\varvec{v}})\varvec{\varepsilon } + (\text {tr}(\varvec{\varepsilon }) - 1) \text {sym}(\nabla {\varvec{v}}) = -\zeta \frac{[N + \eta ^{II}M]c}{1 + a_c^{III} c}\epsilon ,\\ \frac{\text {D}\varvec{\varepsilon }^T}{\text {D}t} + \varvec{\varepsilon }^T \, \text {skw}(\nabla {\varvec{v}}) - \text {skw}(\nabla {\varvec{v}})\varvec{\varepsilon }^T + (\text {tr}(\varvec{\varepsilon }) - 1)\text {sym}(\nabla {\varvec{v}}) = -\zeta \frac{[N + \eta ^{II}M]c}{1 + a_c^{III} c}\epsilon ^T. \end{aligned} \end{aligned}$$

Note that we used $\text {sym}(\nabla {\varvec{v}})^T = \text {sym}(\nabla {\varvec{v}})$ and $\text {skw}(\nabla {\varvec{v}})^T = -\text {skw}(\nabla {\varvec{v}})$. Subtraction gives

17 $$\begin{aligned} \frac{\text {D}}{\text {D}t}(\varvec{\varepsilon } - \varvec{\varepsilon }^T) + (\varvec{\varepsilon } - \varvec{\varepsilon }^T) \, \text {skw}(\nabla {\varvec{v}}) - \text {skw}(\nabla {\varvec{v}})(\varvec{\varepsilon } - \varvec{\varepsilon }^T) = -\zeta \frac{[N + \eta ^{II}M]c}{1 + a_c^{III}c}(\varvec{\varepsilon } - \varvec{\varepsilon }^T). \end{aligned}$$

From the above equation, it is clear that $(\varvec{\varepsilon } - \varvec{\varepsilon }^T) = 0$ represents an equilibrium, and hence symmetry of $\varvec{\varepsilon }$ represents an equilibrium. Hence, we conclude that initial symmetry implies no symmetry changes for later times.

Furthermore, we also prove that $\varvec{\varepsilon } - \varvec{\varepsilon }^T$ is the only solution if $\varvec{\varepsilon } - \varvec{\varepsilon }^T = 0$ at $t = 0$.

Performing the matrix scalar product ${\varvec{A}}:{\varvec{B}}:= \sum _{i,j} A_{ij} B_{ij}$ on the above equation by $\varvec{\varepsilon } - \varvec{\varepsilon }^T$ gives upon setting ${\varvec{w}} = \varvec{\varepsilon } - \varvec{\varepsilon }^T$ and ${\varvec{M}} = \nabla {\varvec{v}}$:

18 $$\begin{aligned} {\varvec{w}}: \frac{\text {D}}{\text {D}t} \, {\varvec{w}} \, + \, {\varvec{w}}: ({\varvec{w}} \, \text {skw}({\varvec{M}})) - {\varvec{w}}: (\text {skw}({\varvec{M}}) \, {\varvec{w}}) = -\zeta \frac{[N + \eta ^{II}M]c}{1 + a_c^{III}c} \, {\varvec{w}}: {\varvec{w}}. \end{aligned}$$

Using $${\varvec{L}} = \text {skw}(\nabla {\varvec{v}}) = \text {skw}({\varvec{M}}) = L_{12} \begin{bmatrix} 0&{}1\\ -1&{}0 \end{bmatrix} (L_{12} = M_{12} - M_{21})$$ and $${\varvec{w}} = \varvec{\varepsilon } - \varvec{\varepsilon }^T = (\varepsilon _{12} - \varepsilon _{21}) \begin{bmatrix} 0&{}1\\ -1&{}0 \end{bmatrix}$$, gives, although ${\varvec{w}}$ and $\text {skw}({\varvec{L}})$ do not commute, that

19 $$\begin{aligned} {\varvec{w}}: ({\varvec{w}} {\varvec{L}}) = 0 \quad \text {and}\quad {\varvec{w}}: ({\varvec{L}} {\varvec{w}}) = 0. \end{aligned}$$

Hence we obtain

20 $$\begin{aligned} {\varvec{w}}: \frac{\text {D}}{\text {D}t} \, {\varvec{w}} = -\zeta \frac{[N + \eta ^{II}M]c}{1 + a_c^{III}c}\,{\varvec{w}}: {\varvec{w}}. \end{aligned}$$

Define $||{\varvec{w}}||^2:= {\varvec{w}}: {\varvec{w}}$, then it follows that

21 $$\begin{aligned} \frac{1}{2} \frac{\text {D}}{\text {D}t} \, ||{\varvec{w}}||^2 = -\zeta \frac{[N + \eta ^{II}M]c}{1 + a_c^{III}c} \, ||{\varvec{w}}||^2. \end{aligned}$$

Integrating over *t* from $t = 0$ and using ${\varvec{w}} = 0$ at $t = 0$, gives

22 $$\begin{aligned} 0 \le ||{\varvec{w}}||^2 = - \zeta \int _0^t \frac{[N + \eta ^{II}M]c}{1 + a_c^{III}c} \, ||{\varvec{w}}||^2 \, \textrm{d}s \le 0. \end{aligned}$$

With $\zeta , N, \eta ^{II}, M, c, a_c^{III} \ge 0$, this implies that $||{\varvec{w}}|| = 0$ on $t > 0$ if $||{\varvec{w}}|| = 0$ on $t = 0$. Hence ${\varvec{w}} = 0$ for $t > 0$, which represents symmetry, is the only possibility if ${\varvec{w}} = 0$ on $t = 0$. $\square$

### Remark 1

Equation (7) depends on a linear relationship between stress and strain, hence $\varvec{\varepsilon } - \varvec{\varepsilon }^T = 0$ is a solution. However, this solution is not guaranteed to be unique; therefore, initial symmetry may change over time because of computing and rounding errors, for instance.

Theorem 1 motivates why we only need to consider $\varepsilon _{12}$ as a cross term assuming initial symmetry. Further, we demonstrate that small perturbations around symmetry of $\varvec{\varepsilon }$ remain small, which is a characteristic of stability.

### Theorem 2

Let Eq. (7) hold on an open Lipschitz domain $\Omega$ for $t>0$. Let $\varvec{\varepsilon }$ be symmetric for $t\ge 0$, then stability of symmetry is warranted if and only if $K \ge 0$.

### Proof

Let $\varvec{\omega } = \varvec{\varepsilon } - \varvec{\varepsilon }^T$ in Eq. (17), then

23 $$\begin{aligned} \frac{\text {D}\varvec{\omega }}{\text {D}t} + \varvec{\omega }\,\text {skw}(\nabla {\varvec{v}}) - \text {skw}(\nabla {\varvec{v}}) \, \varvec{\omega } + K\varvec{\omega } = {\varvec{0}}. \end{aligned}$$

Write $\text {skw}(\nabla {\varvec{v}}) = {\textbf{L}}$, where  skew-symmetric (for any ${\varvec{v}}\in C^2(\Omega )$). Then

24 $$\begin{aligned} \frac{\text {D}\varvec{\omega }}{\text {D}t} + \varvec{\omega }\cdot {\textbf{L}} - {\textbf{L}}\cdot \varvec{\omega } + K\varvec{\omega } = {\varvec{0}}, \end{aligned}$$

a system of ordinary differential equations. Writing out, then

25 $$\begin{aligned} \frac{\text {D}}{\text {D}t}\begin{bmatrix}\omega _{11}&{}\omega _{12}\\ \omega _{21}&{}\omega _{22}\end{bmatrix} - L_{12}\begin{bmatrix}\omega _{12}+\omega _{21}&{}\omega _{22}-\omega _{11}\\ \omega _{22}-\omega _{11}&{}-\omega _{21}-\omega _{12}\end{bmatrix} + K\begin{bmatrix}\omega _{11}&{}\omega _{12}\\ \omega _{21}&{}\omega _{22}\end{bmatrix} = {\varvec{0}}. \end{aligned}$$

Hence

26 $$\begin{aligned} {\left\{ \begin{array}{ll} \frac{\text {D}}{\text {D}t}\omega _{11} - L_{12}(\omega _{12}+\omega _{21}) + K\omega _{11} = 0,\\ \frac{\text {D}}{\text {D}t}\omega _{12} - L_{12}(\omega _{22}-\omega _{11}) + K\omega _{12} = 0,\\ \frac{\text {D}}{\text {D}t}\omega _{21} - L_{12}(\omega _{22}-\omega _{11}) + K\omega _{21} = 0,\\ \frac{\text {D}}{\text {D}t}\omega _{22} + L_{12}(\omega _{21}+\omega _{12}) + K\omega _{22} = 0. \end{array}\right. } \end{aligned}$$

In matrix–vector form, let $\varvec{\omega }=[\omega _{11},\omega _{12},\omega _{21},\omega _{22}]'$, then we get

27 $$\begin{aligned} \frac{\text {D}\varvec{\omega }}{\text {D}t} + {\mathcal {B}}\varvec{\omega } = {\varvec{0}}, \end{aligned}$$

where

28 $$\begin{aligned} {\mathcal {B}} = \begin{bmatrix} K &{} -L_{12} &{} -L_{12} &{} 0\\ L_{12} &{} K &{} 0 &{} -L_{12}\\ L_{12} &{} 0 &{} K &{} -L_{12}\\ 0 &{} L_{12} &{} L_{12} &{} K \end{bmatrix}. \end{aligned}$$

For $K=0$ (in equilibrium, $c=0$ in Eq. (17)), this matrix is skew-symmetric (that is ${\mathcal {B}}^T=-{\mathcal {B}}$), and hence the eigenvalues are zero or purely imaginary. This implies that $\varvec{\omega }=0\Leftrightarrow \varvec{\varepsilon }=\varvec{\varepsilon }^T$ is a null-stable equilibrium. Hence, small perturbations around the symmetry of $\varvec{\varepsilon }$ will remain small, which implies stability. For the case that $K > 0$, it follows that the real part of the eigenvalues is given by *K*, which also gives A-stability (perturbations from symmetry vanish as $t \longrightarrow \infty$). For $K<0$, which corresponds to expansion (instead of contraction in our model), the negative real part of the eigenvalues results in instability of symmetry. Although the current case is not similar to diffusional growth, it is known that diffusional growth in combination with surface processes suffers from Mullins-Sekerka instabilities (Caroli et al. 1986), which exhibits growth of perturbations on spherical surfaces. $\square$

### Remark 2

Of course ${\varvec{v}}$ is non-constant. The only thing that happens is that ${\varvec{v}}={\varvec{v}}(t)$ impacts the angular frequency around the equilibrium $\varvec{\varepsilon }=\varvec{\varepsilon }^T$.

### Stability of the continuous problem

We write the variations around the equilibria in terms of a complex Fourier series,

29 $$\begin{aligned} {\hat{z}}({\textbf{x}},t) = \frac{1}{|\Omega |}\sum _{j,k \in {\mathbb {Z}}} c^z_{j,k}(t)e^{2 i \pi j x}e^{2 i \pi k y}, \end{aligned}$$

for $z\in \{{\hat{N}},{\hat{M}},{\hat{c}},{\hat{\rho }},{\hat{v}}_1,{\hat{v}}_2,{\hat{\varepsilon }}_{11},{\hat{\varepsilon }}_{12},{\hat{\varepsilon }}_{22}\}$, where $|\Omega |$ denotes the measure of $\Omega$ and *i* represents the unit imaginary number.

Substitution of the variations (29) into the linearised equations (13)–(15), multiplication by $e^{-2i\pi l x}e^{-2i\pi py}$, integration over $\Omega =(0,1)^2$ (|$\Omega$|=1) and double orthonormality over $\Omega$ gives

30 $$\begin{aligned} {\dot{c}}^N_{l,p}(t) + {\overline{N}} \left[ (2\pi l)^2 + (2\pi p)^2\right] \left[ D_F c_{l,p}^N(t) - \chi _F c_{l,p}^c(t)\right] + r_F \kappa _F {\overline{N}}^{1+q} c_{l,p}^M(t) \,\nonumber \\ -r_F {\overline{N}}^q \left[ (1 + q)(1 - \kappa _F {\overline{N}}) - \kappa _F {\overline{N}}\right] c_{l,p}^N(t) + \delta _N c_{l,p}^N(t) \,\nonumber \\ -{\overline{N}} \left[ \frac{r_F r^{\text {max}}}{a_c^{I}}(1 - \kappa _F {\overline{N}}) {\overline{N}}^q - k_F\right] c_{l,p}^c(t) = 0,\nonumber \\ {\dot{c}}^M_{l,p}(t) + D_F{\overline{N}}\left[ (2\pi l)^2 + (2\pi p)^2\right] c^M_{l,p}(t) + \delta _M c^M_{l,p}(t) - k_F{\overline{N}} c^c_{l,p}(t) = 0,\nonumber \\ {\dot{c}}^c_{l,p}(t) + D_c\left[ (2\pi l)^2 + (2\pi p)^2\right] c^c_{l,p}(t) + {\overline{N}}\left[ \delta _c{\overline{\rho }} - \frac{k_c}{a_c^{II}} \right] c^c_{l,p}(t) = 0,\nonumber \\ {\dot{c}}^{\rho }_{l,p}(t) +\delta _\rho {\overline{\rho }}^2(\eta ^{II} - \eta ^I) c^M_{l,p}(t) - \delta _\rho {\overline{\rho }}^2{\overline{N}} \left[ \frac{k_\rho ^{max}}{a_c^{IV}} + a_c^{III}\right] c^c_{l,p}(t)\nonumber \\ + 2\delta _\rho {\overline{N}}{\overline{\rho }} c^{\rho }_{l,p}(t) = 0, \end{aligned}$$

for the chemical part of the model, and

31 $$\begin{aligned} \begin{aligned} \rho _t {\dot{c}}_{l,p}^{v_1}(t) + \left[ (2\pi l)^2 (\mu _1 + \mu _2) + \tfrac{1}{2} (2\pi p)^2\mu _1\right] c^{v_1}_{l,p}(t) + (2\pi l)(2\pi p)(\tfrac{1}{2}\mu _1 + \mu _2) c^{v_2}_{l,p}(t) \, \\ -i(2\pi )\left[ \frac{E\sqrt{{\overline{\rho }}}}{1 + \nu }\left\{ p c_{l,p}^{\varepsilon _{12}}(t) + \frac{1 - \nu }{1 - 2\nu } l c_{l,p}^{\varepsilon _{11}}(t) + \frac{\nu }{1 - 2\nu } l c_{l,p}^{\varepsilon _{22}}(t) \right\} + \xi \frac{{\overline{\rho }}}{R^2 + {\overline{\rho }}^2} l c_{l,p}^M(t) \right] \\ -i(2\pi l)\frac{E}{2\sqrt{{\overline{\rho }}}(1 + \nu )}\left[ \overline{\varepsilon _{12}} + \overline{\varepsilon _{11}} + \frac{\nu }{1 - 2\nu }\left( \overline{\varepsilon _{11}} + \overline{\varepsilon _{22}}\right) \right] c_{l,p}^\rho (t) = 0, \end{aligned}\nonumber \\ \end{aligned}$$

for the displacement velocity, and

32 $$\begin{aligned} \begin{aligned} {\dot{c}}_{l,p}^{\epsilon _{11}}(t) + i(2\pi ) \left\{ \left[ l(\overline{\epsilon _{11}} + \overline{\epsilon _{22}} - 1) - p\overline{\epsilon _{12}}\right] c_{l,p}^{v_1}(t) + l\overline{\epsilon _{12}} c_{l,p}^{v_2}(t)\right\} + \zeta {\overline{N}}\overline{\epsilon _{11}}c_{l,p}^c(t) = 0,\\ {\dot{c}}_{l,p}^{\epsilon _{12}}(t) + i(2\pi )\left[ p(\overline{\epsilon _{11}} - \tfrac{1}{2}) c_{l,p}^{v_1}(t) + l(\overline{\epsilon _{22}} - \tfrac{1}{2}) c_{l,p}^{v_2}(t)\right] + \zeta {\overline{N}}\overline{\epsilon _{12}} c_{l,p}^c(t) = 0, \end{aligned}\nonumber \\ \end{aligned}$$

for the effective strain.

Interchanging the third and first equation of (30), these equations together with Eqs. (31) and (32) are in the form $y' +A y = 0$ (*y* the vector of the time-dependent components) with

33 $$\begin{aligned} A = \begin{bmatrix} A_{11} &{}0 &{}0 &{}0 &{}0 &{}0 &{}0 &{}0&{}0\\ A_{21} &{}A_{22} &{}0 &{}0 &{}0 &{}0 &{}0 &{}0&{}0\\ A_{31} &{}A_{32} &{}A_{33} &{}0 &{}0 &{}0 &{}0 &{}0&{}0\\ A_{41} &{}A_{42} &{}0 &{}A_{44} &{}0 &{}0 &{}0 &{}0&{}0\\ 0 &{}A_{52} &{}0 &{}A_{54} &{}A_{55} &{}A_{56} &{}A_{57} &{}A_{58}&{}A_{59}\\ 0 &{}A_{62} &{}0 &{}A_{64} &{}A_{65} &{}A_{66} &{}A_{67} &{}A_{68}&{}A_{69}\\ A_{71} &{}0 &{}0 &{}0 &{}A_{75} &{}A_{76} &{}0 &{}0&{}0\\ A_{81} &{}0 &{}0 &{}0 &{}A_{85} &{}A_{86} &{}0 &{}0&{}0\\ A_{91} &{}0 &{}0 &{}0 &{}A_{95} &{}A_{96} &{}0 &{}0&{}0\\ \end{bmatrix}. \end{aligned}$$

We determine the eigenvalues of *A* by solving $|A - \lambda I|=0$ for $\lambda$, where *I* represents the identity matrix. First, we perform Gaussian elimination to see that we can use the first four diagonal values as pivots. Hence, the first four eigenvalues are the first four diagonal entries. The system is linearly stable if and only if the real part of all the eigenvalues is non-negative, hence stability for the chemical part of the model is guaranteed if:

34 $$\begin{aligned} \begin{aligned} D_F{\overline{N}}\left[ (2\pi l)^2 + (2\pi p)^2\right] - r_F{\overline{N}}^q((1 + q)(1 - \kappa _F{\overline{N}}) - \kappa _F{\overline{N}}) + \delta _N \ge 0,\\ D_F{\overline{N}}\left[ (2\pi l)^2 + (2\pi p)^2\right] + \delta _M \ge 0,\\ D_c\left[ (2\pi l)^2 + (2\pi p)^2\right] + {\overline{N}}\left[ \delta _c{\overline{\rho }} - \frac{k_c}{a_c^{II}}\right] \ge 0,\\ 2\delta _\rho {\overline{N}}{\overline{\rho }} \ge 0. \end{aligned}\nonumber \\ \end{aligned}$$

These four requirements show that stability for the chemical part of the model is equal to the stability constraints in ${\mathbb {R}}^1$ (Egberts et al. 2021b). That is, for $\delta _c\ge \frac{k_c}{a_c^{II}{\overline{\rho }}}$ and $q\delta _N \le \kappa _F r_F{\overline{N}}^{1+q}$ ($l=p=0$). We note we need $\delta _N>0$ and hence $\kappa _F {\overline{N}}<1$. The second and fourth eigenvalues meet the stability condition independent of the chosen parameter values, given that these values are positive. Hence, if the conditions are met for $l = p = 0$, they hold for all $l, p \in {\mathbb {Z}}$, corresponding to wavelike perturbations.

Further, we end up with a $5 \times 5$-submatrix containing the mechanical part of the model. For this $5 \times 5$-matrix, we see that the last three columns contain possibly non-zero values at the first two row positions; hence, these columns are linearly dependent. From this, it immediately follows that $\lambda = 0$ is an eigenvalue.

Applying Gershgorin’s Theorem leads to eigenvalues that can be located anywhere in a union of circles centered around the origin. Hence, Gershgorin’s Theorem does not exclude eigenvalues with a negative real part (which could reside in the left half of the complex plane). For this reason, linear stability for the effective strain is guaranteed if

35 $$\begin{aligned} \begin{aligned} |\alpha |(\overline{\varepsilon _{11}} + \overline{\varepsilon _{12}} + \overline{\varepsilon _{22}} - 1) - |\beta |\overline{\varepsilon _{12}} = 0,\\ |\alpha |(\overline{\varepsilon _{11}} - \tfrac{1}{2}) + |\beta |(\overline{\varepsilon _{22}} - \tfrac{1}{2}) = 0, \end{aligned} \end{aligned}$$

for all $\alpha , \beta \in {\mathbb {Z}}$. Hence, linear stability for wavelike perturbations around equilibria ($\alpha , \beta \in {\mathbb {Z}}_{\ne 0}$) is certainly obtained for $\overline{\varepsilon _{11}} = \tfrac{1}{2}$, $\overline{\varepsilon _{22}} = \tfrac{1}{2}$ and $\overline{\varepsilon _{12}} = 0$. This implies that $A_{i,j} = 0$ for $i \in \{7, 8, 9\}, j \in \{5, 6\}$. We note that with these equilibria, we have $\varvec{\varepsilon } \rightarrow {\mathcal {O}}(1)$, hence describing a physical situation in which the model can no longer be applied. In this case, Gershgorin’s theorem cannot be used to access the stability criteria, and there are no other strategies to solve the fourth-order polynomial analytically. In order to ‘show’ that we have stability for our set of parameter values, we provide an empirical argument based on the numerical approximation of the eigenvalues.

The remaining eigenvalues follow from the $5 \times 5$-matrix. The eigenvalue $\lambda = 0$ has algebraic multiplicity 3. The other two eigenvalues follow from the upper left $2 \times 2$-block matrix and are in addition to that determined by

36 $$\begin{aligned} \lambda ^2 - (A_{55} + A_{66})\lambda + A_{55}A_{66} - A_{56}A_{65} = 0. \end{aligned}$$

We note that $A_{56} = A_{65}$. Hence, the remaining eigenvalues are real-valued. Solving the above equation with the *abc*-formula then gives

37 $$\begin{aligned} \lambda = \frac{A_{55} + A_{66}\pm \sqrt{(A_{55} + A_{66})^2 - 4(A_{55}A_{66} - A_{56}^2)}}{2}. \end{aligned}$$

Here, the discriminant

38 $$\begin{aligned} D= & {} \left[ (2\pi l)^2 - (2\pi p)^2 \right] ^2 (\mu _1 + \mu _2)^2 + \tfrac{1}{4}\left[ (2\pi l)^2 - (2\pi p)^2\right] ^2 \mu _1^2 \, \nonumber \\{} & {} + 4(2\pi l)^2 (2\pi p)^2 \left( \tfrac{1}{2}\mu _2 + \mu _2\right) ^2 \end{aligned}$$

is always non-negative. For stability, in this case, a necessary condition is that

39 $$\begin{aligned} A_{55} + A_{66}\ge \sqrt{(A_{55} + A_{66})^2 - 4(A_{55}A_{66} - A_{56}^2)}. \end{aligned}$$

Squaring the left- and the right-hand-side gives

40 $$\begin{aligned} A_{55}A_{66} - A_{56}^2 \ge 0. \end{aligned}$$

Substitution of $A_{55}, A_{66}$ and $A_{56}$ gives

41 $$\begin{aligned}{} & {} - \frac{4}{\rho _t} \left( \frac{1}{\rho _t}\left[ (2\pi l)^2 (\mu _1 + \mu _2) + \tfrac{1}{2}(2\pi p)^2 \mu _1\right] \cdot \left[ \tfrac{1}{2}(2\pi l)^2 \mu _1 + (2\pi p)^2 (\mu _1 + \mu _2)\right] \right. \nonumber \\{} & {} \quad - \left. ((2\pi l)(2\pi p)(\tfrac{1}{2}\mu _1 + \mu _2))^2\right) \le 0, \end{aligned}$$

which reduces to

42 $$\begin{aligned} \left[ \tfrac{1}{2}((2\pi l)^4 + (2\pi p)^4) + (2\pi l)^2 (2\pi p)^2\right] (\mu _1^2 + \mu _1\mu _2 )\ge 0. \end{aligned}$$

Hence, for all $\mu _1, \mu _2\ge 0$ and all $l, p \in {\mathbb {Z}}$, the stability constraint is satisfied. We summarize these results in Theorem 3.

#### Theorem 3

Let $\{N, M, c, \rho , v_1, v_2, \varepsilon _{11}, \varepsilon _{12}, \varepsilon _{22}\}$ satisfy Equations (1)–(7). Let $\delta _N = r_F (1 - \kappa _F {\overline{N}}) {\overline{N}}^{q} > 0$ and ${\overline{\rho }} = \sqrt{k_\rho /\delta _\rho }$, then

1. The equilibria $(N, M, c, \rho , v_1, v_2, \varepsilon _{11}, \varepsilon _{12}, \varepsilon _{22}) = ({\overline{N}}, 0, 0, {\overline{\rho }}, 0, 0, \overline{\varepsilon _{11}}, \overline{\varepsilon _{12}}, \overline{\varepsilon _{22}})$, with $\{{\overline{N}}, {\overline{\rho }}, \overline{\varepsilon _{11}}, \overline{\varepsilon _{12}}, \overline{\varepsilon _{22}}\} \in {\mathbb {R}}_{>0}$, are linearly stable if and only if $\delta _c{\overline{\rho }} \ge \frac{k_c}{a_c^{II}}$, and $q\delta _N \le \kappa _F r_F{\overline{N}}^{1+q}$ for constant states;
2. For (nonconstant) waves around the equilibria, linear stability is met if $\delta _c{\overline{\rho }}\ge \frac{k_c}{a_c^{II}}$, $q\delta _N \le \kappa _F r_F{\overline{N}}^{1+q}$, $\mu _1, \mu _2 \ge 0$, $\overline{\varepsilon _{11}} = \overline{\varepsilon _{22}} = \tfrac{1}{2}$, and $\overline{\varepsilon _{12}} = 0$;

#### Remark 3

Note that $\delta _c \ge \frac{k_c}{a_c^{II}{\overline{\rho }}}$, for $k=0$ (constant states). Hence, if constant perturbations are stable, then wavelike perturbations are stable. In case $\delta _c$ is not large enough, fast oscillating perturbations will vanish, while slow oscillating perturbations will not vanish and can amplify. Further, the mathematical model is actually not suitable for $\overline{\varepsilon _{11}} = \overline{\varepsilon _{22}} = 0.5$; however, this is still a consequence of the above analysis.

For the empirical ‘proof’ of the stability constraints, we only consider the eigenvalues $\varvec{\lambda }$ of the $5 \times 5$-submatrix of matrix *A*. We keep the parameter values as in Table2 and vary $\overline{\varepsilon _{11}}$ and $\overline{\varepsilon _{22}}$ between $-$ 1 and 1 with stepsize 0.01, $\overline{\varepsilon _{12}}$ between $-$ 1 and 0.5 with stepsize 0.1, and *l* and *p* (integers) between 1 and 100. We define

43 $$\begin{aligned} S(\overline{\varepsilon _{11}}, \overline{\varepsilon _{22}}, \overline{\varepsilon _{12}}) = {\left\{ \begin{array}{ll} 1, \quad \text {if} \, \forall \, l, p \in {\mathbb {Z}}: \, Re(\varvec{\lambda }) \ge {\varvec{0}}\\ 0, \quad \text {otherwise} \end{array}\right. }. \end{aligned}$$

**Fig. 1:**
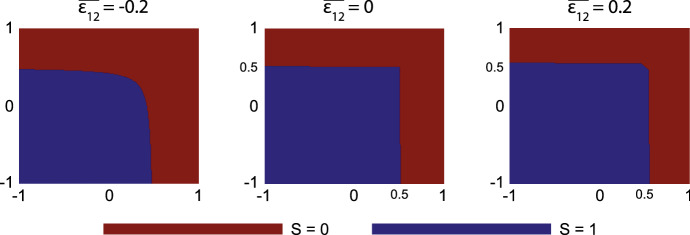
Results of the empirical proof of stability for some values of $\overline{\varepsilon _{12}}$. The *x*- and *y*-axes show the effective strains $\overline{\varepsilon _{11}}$ and $\overline{\varepsilon _{22}}$, both between $-$ 1 and 1. Values for $\overline{\varepsilon _{11}}$ and $\overline{\varepsilon _{22}}$ that yield $Re(\varvec{\lambda }) \ge {\varvec{0}}$ for all *l*, *p* are colored blue, otherwise red

Hence, $S = 1$ corresponds to stability, whereas $S = 0$ corresponds to instability. Figure 1 shows the results of *S* for some values of $\overline{\varepsilon _{12}}$.

If $\overline{\varepsilon _{12}}$ increases, then the region for stable $\overline{\varepsilon _{11}}$ and $\overline{\varepsilon _{22}}$ values grows. Further, there is symmetry in the line $y = x$. Given the complexity of the fourth-order polynomial, it is unclear what defines these boundaries of stable regions. One can predict that there will not be any stable values for the effective strain in the shown domain if $\overline{\varepsilon _{12}}$ becomes (much) smaller. However, one does not expect the effective strain to become that negative (the model breaks down for these values). Focusing on the origin $(\overline{\varepsilon _{11}}, \overline{\varepsilon _{22}}) = (0, 0)$, one can see that for small $|\overline{\varepsilon _{12}}|$, say $|\overline{\varepsilon _{12}}| \le 0.2$, there is always a stable region around the origin. For values of $|\overline{{\varepsilon }_{12}}|$ that are larger, which are not interesting from a physical point of view, the stability region may no longer include the origin.

#### Claim

Let $\{N, M, c, \rho , v_1, v_2, \varepsilon _{11}, \varepsilon _{12}, \varepsilon _{22}\}$ satisfy Eqs. (1)–(7). Let $\delta _N = r_F (1 - \kappa _F {\overline{N}}) {\overline{N}}^{q} > 0$ and ${\overline{\rho }} = \sqrt{k_\rho /\delta _\rho }$, then for wave-like perturbations around the equilibria, if $\delta _c{\overline{\rho }} \ge \frac{k_c}{a_c^{II}}$, $q\delta _N \le \kappa _F r_F{\overline{N}}^{1+q}$, and $\mu _1, \mu _2 \ge 0$, then there is a region in the $(\overline{\varepsilon _{11}}, \overline{\varepsilon _{12}}, \overline{\varepsilon _{22}})$–space around $(\overline{\varepsilon _{11}}, \overline{\varepsilon _{12}}, \overline{\varepsilon _{22}}) = (0, 0, 0)$ (the origin), where the model is linearly stable.

### Stability of the discrete problem

The stability of the continuous problem does not always automatically imply the stability of the discrete counterpart of the problem. Therefore, we assess the stability of the semi-discrete problem, which we use to analyze the stability of the whole discrete system. Lax’s Equivalence Theorem states that a consistent, stable method converges. Under stability, the global truncation error tends to zero as the step size tends to zero (as $h \rightarrow 0$) if the local truncation error (i.e., the difference between the derivatives and difference ratios) tends to zero as the step size goes to zero.

We consider a unit rectangle $\Omega = [0, 1]^2$ that we divide into small rectangles with sides *h* such that $(n+1)h = 1$. At the intersections of the grid lines, we have nodal points where we approximate the solutions of the variables. We denote the unknowns at node $(x_i,y_j)$ by $z_{i,j}$, $z \in \{N, M, c, \rho , v_1, v_2, \varepsilon _{11}, \varepsilon _{12}, \varepsilon _{22}$} and apply finite differences on the eigenvalue problem. Then the finite difference method (FDM) of the spatial part of the linearized problem (13)–(15) gives:44for the chemical part of the model, and writing $v^1_{i,j}$ for ${v_1}_{i,j}$ and $\varepsilon ^{11}_{i,j}$ for ${\varepsilon _{11}}_{i,j}$ etc. gives

45 $$\begin{aligned} \begin{aligned} \rho _t\lambda v^1_{i,j}&= - \left( \mu _1 + \mu _2\right) \frac{v^1_{i-1,j} - 2v^1_{i,j} + v^1_{i+1,j}}{h^2} - \frac{\mu _1}{2}\frac{v^1_{i,j-1} - 2v^1_{i,j} + v^1_{i,j+1}}{h^2} \\ {}&\quad - \left[ \frac{\mu _1}{2} + \mu _2\right] \frac{v^2_{i-1,j-1} - v^2_{i-1,j+1} - v^2_{i+1,j-1} + v^2_{i+1,j+1}}{4h^2} \\ {}&\quad - \frac{E\sqrt{{\overline{\rho }}}}{1 + \nu }\left[ \frac{\varepsilon ^{12}_{i,j+1} - \varepsilon ^{12}_{i,j-1}}{2h} + \frac{1 - \nu }{1 - 2\nu }\frac{\varepsilon ^{11}_{i+1,j} - \varepsilon ^{11}_{i-1,j}}{2h} + \frac{\nu }{1 - 2\nu }\frac{\varepsilon ^{22}_{i+1,j} - \varepsilon ^{22}_{i-1,j}}{2h} \right] \\ {}&\quad - \frac{E}{2\sqrt{{\overline{\rho }}}(1 + \nu )}\left[ \overline{\varepsilon _{12}} + \overline{\varepsilon _{11}} + \frac{\nu }{1 - 2\nu }\left( \overline{\varepsilon _{11}} + \overline{\varepsilon _{22}}\right) \right] \frac{\rho _{i+1,j} - \rho _{i-1,j}}{2h}\\ {}&\quad - \xi \frac{{\overline{\rho }}}{R^2 + {\overline{\rho }}^2}\frac{M_{i+1,j} - M_{i-1,j}}{2h}, \end{aligned}\qquad \end{aligned}$$

for $v_1$ (again, the equation for $v_2$ is similar), and

46 $$\begin{aligned}{} & {} \lambda \varepsilon ^{11}_{i,j} = \overline{\epsilon _{12}}\left[ \frac{v^2_{i+1,j} - v^2_{i-1,j}}{2h} - \frac{v^1_{i,j+1} - v^1_{i,j-1}}{2h}\right] + (\overline{\epsilon _{11}} + \overline{\epsilon _{22}} - 1)\frac{v^1_{i+1,j} - v^1_{i-1,j}}{2h} \, \nonumber \\{} & {} \quad +\zeta {\overline{N}}\overline{\epsilon _{11}}c_{i,j}, \nonumber \\{} & {} \quad \lambda \varepsilon ^{12}_{i,j} = \left[ \overline{\epsilon _{22}} - \frac{1}{2}\right] \frac{v^2_{i+1,j} - v^2_{i-1,j}}{2h} + \left[ \overline{\epsilon _{11}} - \frac{1}{2}\right] \frac{v^1_{i,j+1} - v^1_{i,j-1}}{2h} + \zeta {\overline{N}}\overline{\epsilon _{12}}c_{i,j}, \nonumber \\ \end{aligned}$$

for the effective strains $\varepsilon _{11},\varepsilon _{12}$ (again, the equation for $\varepsilon _{22}$ is similar). We perform von Neumann eigenvalue and stability analysis. Let

47 $$\begin{aligned} z_{k,j} = \frac{1}{n^2}\sum _{\beta =1}^{n}\sum _{\gamma =1}^{n} {\hat{z}}_{\beta ,\gamma } e^{-2\pi \beta khi}e^{-2\pi \gamma jhi}, \end{aligned}$$

for $z \in \{N, M, c, \rho , v_1, v_2, \varepsilon _{11}, \varepsilon _{12}, \varepsilon _{22}\}$, where *i* again represents the imaginary unit number.

Substitution of (47) in Eqs. (44)–(46), multiplication by $e^{2\pi l khi} e^{2\pi p jhi}$, using Euler’s formula, $2 - 2\cos (2\pi x) = 4\sin ^2(\pi x)$ and $\Big [e^{ix} e^{iy} - e^{ix} e^{-iy} - e^{-ix} e^{iy} + e^{-ix} e^{-iy}\Big ]/4 = -\sin (x)\sin (y)$ results in

48 $$\begin{aligned} \lambda {\hat{N}}_{\varvec{\beta }}&= \frac{4{\overline{N}}}{h^2}\left[ \sin ^2(\pi l h) + \sin ^2(\pi p h)\right] \left[ D_F{\hat{N}}_{\varvec{\beta }} - \chi _F{\hat{c}}_{\varvec{\beta }}\right] + \nonumber \\&\qquad \left[ \delta _N - r_F{\overline{N}}^q((1+q)(1 - \kappa _F{\overline{N}}) - \kappa _F{\overline{N}})\right] {\hat{N}}_{\varvec{\beta }} \,+\nonumber \\&\qquad \quad r_F\kappa _F{\overline{N}}^{1+q} {\hat{M}}_{\varvec{\beta }} - {\overline{N}}\left[ \frac{r_F r^{\text {max}}}{a_c^{I}}(1 - \kappa _F{\overline{N}}){\overline{N}}^q - k_F\right] {\hat{c}}_{\varvec{\beta }},\nonumber \\ \lambda {\hat{M}}_{\varvec{\beta }}&= \left[ \frac{4D_F{\overline{N}}}{h^2}\left[ \sin ^2(\pi l h) + \sin ^2(\pi p h)\right] + \delta _M\right] {\hat{M}}_{\varvec{\beta }} - k_F{\overline{N}}{\hat{c}}_{\varvec{\beta }},\nonumber \\ \lambda {\hat{c}}_{\varvec{\beta }}&= \left[ \frac{4D_c}{h^2}\left[ \sin ^2(\pi l h) + \sin ^2(\pi p h)\right] + {\overline{N}}\left[ \delta _c{\overline{\rho }} - \frac{k_c}{a_c^{II}}\right] \right] {\hat{c}}_{\varvec{\beta }},\nonumber \\ \lambda {\hat{\rho }}_{\varvec{\beta }}&=\delta _\rho {\overline{\rho }}^2(\eta ^{II} - \eta ^I){\hat{M}}_{\varvec{\beta }} - \delta _\rho {\overline{\rho }}^2{\overline{N}}\left[ \frac{k_\rho ^{max}}{a_c^{IV}} + a_c^{III}\right] {\hat{c}}_{\varvec{\beta }} + 2\delta _\rho {\overline{N}}{\overline{\rho }}{\hat{\rho }}_{\varvec{\beta }},\nonumber \\ \end{aligned}$$

for the chemical part of the model and

49 $$\begin{aligned} \begin{aligned} \rho _t\lambda {\hat{v}}^1_{\varvec{\beta }}&=\frac{4}{h^2}\left[ \sin ^2(\pi l h)(\mu _1 + \mu _2) + \tfrac{1}{2}\sin ^2(\pi p h)\mu _1\right] {\hat{v}}^1_{\varvec{\beta }} \, \\&\quad +\frac{\sin (2\pi l h)\sin (2\pi p h)}{h^2}\left( \tfrac{1}{2}\mu _1 + \mu _2\right) {\hat{v}}^2_{\varvec{\beta }} \,\\&\quad + i\frac{E\sqrt{{\overline{\rho }}}}{h(1 + \nu )}\left[ \sin (2\pi p h){\hat{\varepsilon }}^{12}_{\varvec{\beta }} + \sin (2\pi l h)\left( \frac{1 - \nu }{1 - 2\nu }{\hat{\varepsilon }}^{11}_{\varvec{\beta }} + \frac{\nu }{1 - 2\nu }{\hat{\varepsilon }}^{22}_{\varvec{\beta }}\right) \right] + \\&\quad i\frac{\sin (2\pi l h)}{h}\left\{ \frac{E}{2\sqrt{{\overline{\rho }}}(1 + \nu )}\left[ \overline{\varepsilon _{12}} + \overline{\varepsilon _{11}} + \frac{\nu }{1 - 2\nu }\left( \overline{\varepsilon _{11}} + \overline{\varepsilon _{22}}\right) \right] {\hat{\rho }}_{\varvec{\beta }} + \frac{\xi {\overline{\rho }}}{R^2 + {\overline{\rho }}^2}{\hat{M}}_{\varvec{\beta }}\right\} , \end{aligned}\nonumber \\ \end{aligned}$$

for the displacement velocity $v_1$ (the equation for $v_2$ is similar and yields an equal result), and

50 $$\begin{aligned} \begin{aligned} \lambda {\hat{\varepsilon }}^{11}_{\varvec{\beta }}&= i\left\{ \frac{\sin (2\pi l h)}{h}(1 - \overline{\epsilon _{11}} - \overline{\epsilon _{22}}) + \frac{\sin (2\pi p h)}{h}\overline{\epsilon _{12}}\right\} {\hat{v}}^1_{\varvec{\beta }}\\&\quad \quad - i\frac{\sin (2\pi l h)}{h}\overline{\epsilon _{12}}{\hat{v}}^2_{\varvec{\beta }} + \zeta {\overline{N}}\overline{\epsilon _{11}}{\hat{c}}_{\varvec{\beta }},\\ \lambda {\hat{\varepsilon }}^{12}_{\varvec{\beta }}&= i\frac{\sin (2\pi p h)}{h}(\tfrac{1}{2} - \overline{\epsilon _{11}}){\hat{v}}^1_{\varvec{\beta }} + i\frac{\sin (2\pi l h)}{h}(\tfrac{1}{2} - \overline{\epsilon _{22}}){\hat{v}}^2_{\varvec{\beta }} + \zeta {\overline{N}}\overline{\epsilon _{12}}{\hat{c}}_{\varvec{\beta }}, \end{aligned}\nonumber \\ \end{aligned}$$

for the effective strains $\varepsilon _{11}$ and $\varepsilon _{12}$ (the equation for $\varepsilon _{22}$ is similar and yields an equal result for ${\overline{\varepsilon }}_{22}$). As an example, the derivation of Eq. (49) is given in the Appendix.

The Eqs. (48)–(50) are in the form $\lambda z = C z$ with the matrix *C* as in (33). Hence, the eigenvalues are found the same way as in the continuous case. Note that, since the overall system has the form ${\underline{y}}' + A{\underline{y}} = 0$, the discrete system is linearly stable if and only if the real part of the eigenvalues is non-negative, hence we need:

51 $$\begin{aligned} \begin{aligned} \frac{4D_F{\overline{N}}}{h^2}\left[ \sin ^2(\pi l h) + \sin ^2(\pi p h)\right] - r_F{\overline{N}}^q((1 + q)(1 - \kappa _F{\overline{N}}) - \kappa _F{\overline{N}}) + \delta _N \ge 0,\\ \frac{4D_F{\overline{N}}}{h^2}\left[ \sin ^2(\pi l h) + \sin ^2(\pi p h)\right] + \delta _M \ge 0,\\ \frac{4D_c}{h^2}\left[ \sin ^2(\pi l h) + \sin ^2(\pi p h)\right] + {\overline{N}}\left[ \delta _c{\overline{\rho }} - \frac{k_c}{a_c^{II}}\right] \ge 0,\\ 2\delta _\rho {\overline{N}}{\overline{\rho }} \ge 0, \end{aligned}\nonumber \\ \end{aligned}$$

for the chemical part of the model. To guarantee linear stability, the first requirement of Eq. (51) states $q\delta _N \le \kappa _F r_F{\overline{N}}^{1+q}$, given $\delta _N = r_F (1 - \kappa _F {\overline{N}}) {\overline{N}}^{q}$. The second and fourth eigenvalues meet the stability condition independent of the chosen values for the parameters, given that the parameters are positive. Finally, the third requirement states $\delta _c{\overline{\rho }}\ge \frac{k_c}{a_c^{II}}$. These statements remain the same when the horizontal and vertical step sizes are unequal ($\Delta x\ne \Delta y$).

For the mechanical part of the model, we follow the same procedure as in Sect. 3.1. Again, we end up with a $5\times 5$-matrix *D* containing the mechanical part of the model. Now, from Gershgorin (not shown), it follows that $\overline{\epsilon _{11}} = \tfrac{1}{2}, \overline{\epsilon _{12}} = 0$, and $\overline{\epsilon _{22}} = \tfrac{1}{2}$, and $C_{i,j} = 0$ for $i\in \{7, 8, 9\}, j\in \{5, 6\}$. Therefore, for linear stability, we need

52 $$\begin{aligned} C_{55}C_{66} - C_{56}^2 \ge 0. \end{aligned}$$

Substitution of $C_{55}, C_{66}$ and $C_{56}$ gives

53 $$\begin{aligned}{} & {} - 4\left( \frac{4}{\rho _th^2}\left[ \sin ^2(\pi l h)(\mu _1 + \mu _2) + \tfrac{1}{2}\sin ^2(\pi p h)\mu _1\right] \cdot \right. \nonumber \\{} & {} \frac{4}{\rho _th^2}\left[ \tfrac{1}{2}\sin ^2(\pi l h)\mu _1 + \sin ^2(\pi p h)(\mu _1 + \mu _2)\right] - \nonumber \\{} & {} \left. \left[ \frac{1}{\rho _t}\frac{\sin (2\pi l h)\sin (2\pi p h)}{h^2}\left( \mu _1 + \mu _2\right) \right] ^2\right) \le 0, \end{aligned}$$

which reduces to

54 $$\begin{aligned}{} & {} \mu _1^2\left[ 8(\sin ^4(\pi l h) + \sin ^4(\pi p h)) + 4\sin ^2(\pi l h)\sin ^2(\pi p h)\left[ 5 - 2\cos ^2(\pi lh)\cos ^2(\pi ph)\right] \right] +\nonumber \\{} & {} \left. \mu _1\mu _2\left[ 8(\sin ^4(\pi l h) + \sin ^4(\pi p h)) + 16\sin ^2(\pi l h)\sin ^2(\pi p h)\left[ 2 - \cos ^2(\pi lh)\cos ^2(\pi ph)\right] \right] \right. + \nonumber \\{} & {} 8\mu _2^2\sin ^2(\pi l h)\sin ^2(\pi p h)\left[ 2 - \cos ^2(\pi lh)\cos ^2(\pi ph)\right] \ge 0. \end{aligned}$$

Here, we used $\sin (2\pi x h) = 2\sin (\pi x h)\cos (\pi x h)$. Note that the subtractions by the cosines are bounded from above. Therefore, for all $\mu _1, \mu _2$ and all $l, p \in {\mathbb {Z}}$, Eq. (53) is satisfied. To conclude, we have demonstrated that if the equilibrium is stable in the continuous problem, it is also stable in the semi-discrete problem.

A consistency exists between the continuous problem’s stability criteria and the discrete problem’s stability criteria. We show this by writing $\sin (x)$ and $\sin ^2(x)$ as a Taylor series. Substitution into the third equation in (51) yields:

55 $$\begin{aligned} D_c[(2\pi l)^2 + (2\pi p)^2] + {\mathcal {O}}(h^2) + {\overline{N}}\left[ \delta _c{\overline{\rho }} - \frac{k_c}{a_c^{II}} \right] \ge 0. \end{aligned}$$

Comparison to the third equation in (34)

56 $$\begin{aligned} D_c[(2\pi l)^2 + (2\pi p)^2] + {\overline{N}}\left[ \delta _c{\overline{\rho }} - \frac{k_c}{a_c^{II}}\right] \ge 0 \end{aligned}$$

yields a difference in eigenvalues of order ${\mathcal {O}}(h^2)$. Note that this difference of the same order is found for the other eigenvalues. We summarise the results in Theorem 4.

#### Theorem 4

Let $\{N, M, c, \rho , v_1, v_2, \varepsilon _{11}, \varepsilon _{12}, \varepsilon _{22}\}$ satisfy the semi-discrete spatial differences version of Eqs. (44)–(46). Then, the fully continuous problem stability implies the semi-discrete formulation’s stability.

#### Corollary 1

Let $\{N, M, c, \rho , v_1, v_2, \varepsilon _{11}, \varepsilon _{12}, \varepsilon _{22}\}$ satisfy the semi-discrete spatial differences version of Eqs. (44)–(46). Let $\delta _N = r_F (1 - \kappa _F {\overline{N}}) {\overline{N}}^{q}$ and ${\overline{\rho }} = \sqrt{k_\rho /\delta _\rho }$. Then the constant equilibria are unconditionally stable for the trapezoid rule and the Euler backward method as long as $\delta _c{\overline{\rho }} \ge \frac{k_c}{a_c^{II}}$ and $q\delta _N \le \kappa _F r_F{\overline{N}}^{1+q}$. Furthermore, the Euler backward method is A-stable.

## Numerical validation

We need to validate whether the linear stability conditions we have derived also hold in a finite element setting where we consider the fully nonlinearly coupled model. We approximate the solution to the model equations by the finite element method using bi-linear basis functions (Van Kan et al. 2014). We refer to Appendix 2 in Koppenol and Vermolen (2017) for deriving the general finite-element approximation. We consider a rectangle $\Omega = [0,L]^2$ that we subdivide into small rectangles (quadrilaterals) with sides $\Delta x = \Delta y$. Then, we convert the regular mesh to a triangulation. The quadrilateral mesh faces are converted to triangles by splitting the faces into triangles according to a cross-division of the quadrilateral. Let $\Omega \approx \Omega ^h = \bigcup _{e=1}^{n_e}\Omega _e$ the global finite element subspace and ${\textbf{a}}_j, j\in \{1,\dots ,n\}$, $n\in {\mathbb {N}}$ the coordinates of these vertices of the elements. We choose the piecewise linear Lagrangian basis functions $\varphi _i\in X_h(t)$ with $\varphi _i({\textbf{a}}_j,t) = \delta _{ij},\quad i,j\in \{1,\dots ,n\}$ as basis functions for the finite-dimensional subspace $\Omega ^h$, where $\delta _{ij}$ denotes the Kronecker delta function. Note that the following holds for the chosen subspace $\Omega ^h \subset \Omega _{{\textbf{x}},t}$: $\frac{\textrm{D}\varphi _i}{\textrm{D}t} = 0$ for all $\varphi _i$ (Dziuk and Elliot 2007). We simplify the Galerkin equations using this property. We approximate the integrals over the interior of the elements by a Newton-Cotes rule based on linear basis functions.

Regarding the computation of the integrals that arise during the discretization, we apply the Newton-Cotes integration rule and backward Euler time integration. We use a monolithic approach with inner Picard iterations to account for the non-linearity of the model equations. We approximate the local displacements of the dermal layer $({\textbf{u}})$ with

57 $$\begin{aligned} \textbf{u}(\textbf{x}(t+\Delta t), t+\Delta t) \approx \textbf{u}(\textbf{x}(t),t) + \textbf{v}(\textbf{x}(t),t)\Delta t. \end{aligned}$$

Further, we update the mesh and keep track of the mesh quality by computing

$$\begin{aligned} \min _{e_k}\left| {\textbf{J}}_{e_k}\right| /\max _{e_k}\left| {\textbf{J}}_{e_k}\right| ,\quad e_k\in \Omega , \end{aligned}$$

with ${\textbf{J}}$ the Jacobian. In case $\min _{e_k}\left| {\textbf{J}}_{e_k}\right| /\max _{e_k}\left| {\textbf{J}}_{e_k}\right| <0.5$, we perform remeshing. In all simulations, this ratio was at least 0.9915; remeshing was unnecessary. We use mass lumping to avoid loss of monotonicity (i.e., oscillations).

We experimentally evaluate the convergence of the numerical method in a domain of $[0,3.2]^2$ cm$^2$ with a wound between [0, 1.2] cm$^2$. This domain represents a quarter of the domain of the modeled skin on which we perform computations, which is possible because of the model’s symmetry. The transition from healthy to injured skin is steep, and we account for this steepness of gradients through an interval of 0.8 cm. In this transition, the initial solutions vary between the equilibria and the initial wound densities. Within the wound, we assume that 2000 fibroblast cells/cm$^3$, $10^{-8}$ g/cm$^3$ signaling molecules, and 0.01125 g/cm$^3$ collagen are present. We model the slope of the variables with sine functions.

We divide the computation domain into $n_x\times n_y=(3.2/h)^2$ elements with $h\in \{0.2, 0.1, 0.05\}$. In order to have mesh convergence of the numerical solution of the second order, we choose $\Delta t = h^2$. We simulate post-burn contraction for one day and report the densities of the variables (the solutions). We compute the convergence order results using the $L_2$ error norm. Let $\lim _{h\rightarrow 0} z_h({\textbf{x}}, 1) = z({\textbf{x}}, 1)$ denote the true density of variable *z* on day one and $z_{0.05}({\textbf{x}}, 1) =: z_{h_\text {ref}}$ the solution in the last simulation (i.e., the reference, the one computed using the highest numerical resolution). We approximate the errors with the following error definition:

58 $$\begin{aligned} \epsilon _{L^2} (z_h) = h\sqrt{\sum _{i=1}^{289}(z_h({\textbf{x}}_{i,289},1) - z_{h_\text {ref}}({\textbf{x}}_{i,289}, 1))^2}, \end{aligned}$$

where the grid-points ${\textbf{x}}_{i,n}$ correspond to the grid-points in the simulation with $h=0.2$ ($n=289$ nodes). Hence, we evaluate the solution to the equations between simulations on a fixed set of initial nodes.

**Table 1:** Overview of the slopes of the $L_2$ errors of the variables on the total computational domain

Var	*N*	*M*	*c*	$\rho$	$v_1$	$v_2$	$\varepsilon _{11}$	$\varepsilon _{12}$	$\varepsilon _{22}$
$\epsilon _{L_2}$	2.236	2.815	2.489	2.877	2.923	2.923	2.230	2.276	2.230

The reference is the solution in which $h = 0.05$

Table 1 shows the results for the $L_2$ error. All the $L_2$ errors decrease consistently as *h* becomes smaller (figures not shown), and the values in the table show an order of convergence above ${\mathcal {O}}(h^2)$.

We perturb the initial conditions around equilibria using sine functions and vary the parameter $\delta _c$. Hence, we fix all parameters except for the signaling molecule decay rate. Table 2 shows the fixed parameter values. For the time integration, we use an initial step of $\Delta t = 10^{-2}$ days until half a day is simulated, after which we increase the time step by $\Delta t_{\text {new}} = \min \{2,1.1\times \Delta t\}$. We use a domain of $[0,1]^2$ cm$^2$ that we first divide into equilateral elements (rectangles) with $h=0.05$; then, we convert this mesh to an equilateral triangulation by cross division of quadrilaterals.

For the initial conditions, we vary the number of waves *k* using two levels (1 and 2). We perturb the initial condition for the fibroblasts and collagen by using a product of sine functions with an amplitude of 10 cells/cm$^3$ and $10^{-2}$ g/cm$^3$, respectively. Since the fibroblasts’ and collagen’s biological equilibria are non-zero, we do not need to worry about negative values in these initial conditions. For the initial condition of the myofibroblasts and the signaling molecules, we use a product of uniform splines with $2k+1$ knots. On the boundaries, the knots have zero value, and between the values are 3 and 1 cells/cm$^3$ for the myofibroblasts and $2 \times 10^{-7}$ and $5\times 10^{-8}$ g/cm$^3$ for the signaling molecules. These setups ensure that the myofibroblast distribution and the signaling molecule density values are positive. We note that these initial amplitudes become smaller because of the product of splines. The initial amplitudes of the displacement velocity $v_1$ is $5\times 10^{-1}$. To ensure symmetry, we set the initial condition $v_2({\textbf{x}},0)=-v_1({\textbf{x}},0)$. We do not perturb the effective strain densities and set $\varepsilon _{11}({\textbf{x}},0) = \varepsilon _{12}({\textbf{x}},0) = \varepsilon _{22}({\textbf{x}},0) = 0$.

For stability, Theorem 3 further requires that $\delta _c{\overline{\rho }} \ge \frac{k_c}{a_c^{II}}$ in case $l = 0$. We choose to vary the signaling molecule decay rate $\delta _c$ using three levels: $2\times 10^{-4}, 3\times 10^{-4}$ and $5\times 10^{-4}$ cm$^6$/(cells g day), where the first two values do not meet the stability condition.

**Fig. 2:**
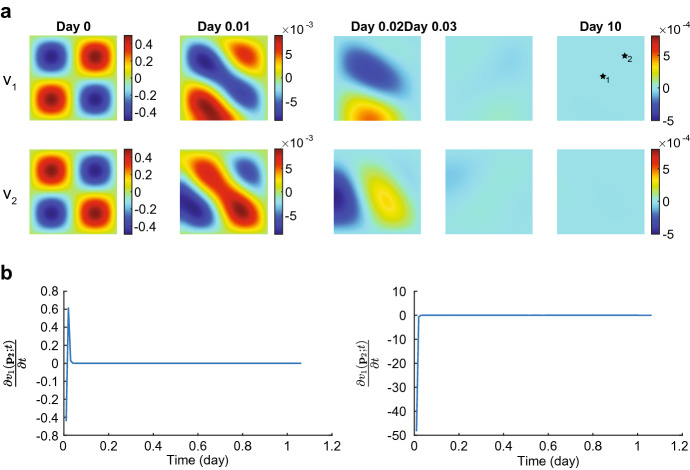
Evolution of the displacement velocity densities for $\delta _c = 5\times 10^{-4}$ cm$^6$/(cells g day). Table 2 shows the values of the other parameters. In subfigure **a** the upper plots show the displacement velocity $v_1$, and the lower plots show the displacement velocity $v_2$. The shown domains are $(0,1)^2$ cm$^2$, and the color bars show the displacement velocities in cm/day. For both $v_1$ and $v_2$, the first two plots have different color bars, and the last three plots share the same color bar shown on the right. In subfigure **b** the left and right plots show the time-dependent change of $v_1$ at points ${\textbf{p}}_1\approx (0.5,0.5)$ and ${\textbf{p}}_2\approx (0.75,0.75)$, respectively (see the black stars in the last plot for $v_1$ in (**a**). Note that these points are subject to displacement

**Fig. 3:**
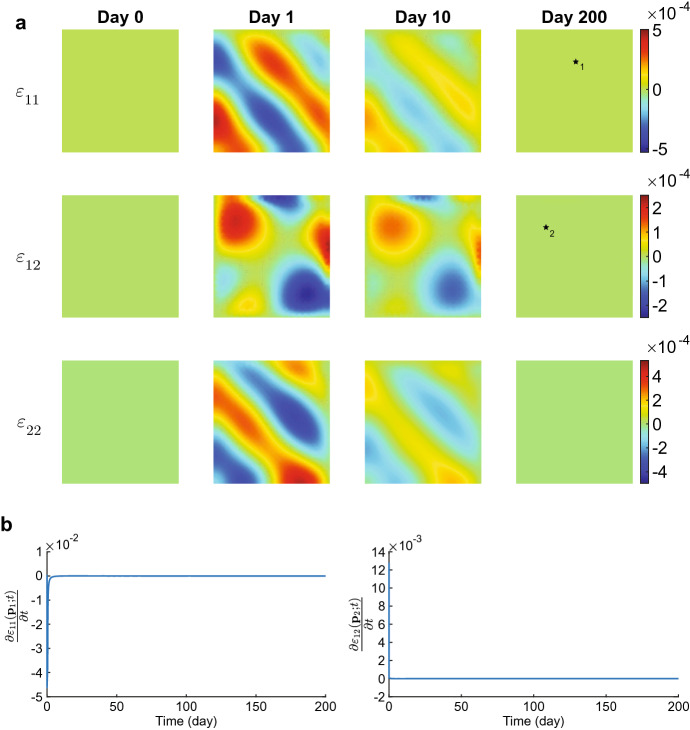
Evolution of the effective strain densities for $\delta _c = 5\times 10^{-4}$ cm$^6$/(cells g day). Table 2 shows the values of the other parameters. In subfigure **a** the upper, middle, and lower plots show the effective strains $\varepsilon _{11}, \varepsilon _{12}$, and $\varepsilon _{22}$, respectively. The shown domains are $(0,1)^2$ cm$^2$. The color bars show the effective strains (no unit) and correspond to the four plots shown on the left of the color bars. In subfigure **b** the left and right plots show the time-dependent change of $\varepsilon _{11}$ at point ${\textbf{p}}_1\approx (0.5,0.75)$ and of $\varepsilon _{12}$ at point ${\textbf{p}}_2\approx (0.25,0.75)$, respectively (see the black stars in the last plots for $\varepsilon _{11}$ and $\varepsilon _{12}$ in (**a**)

**Fig. 4:**
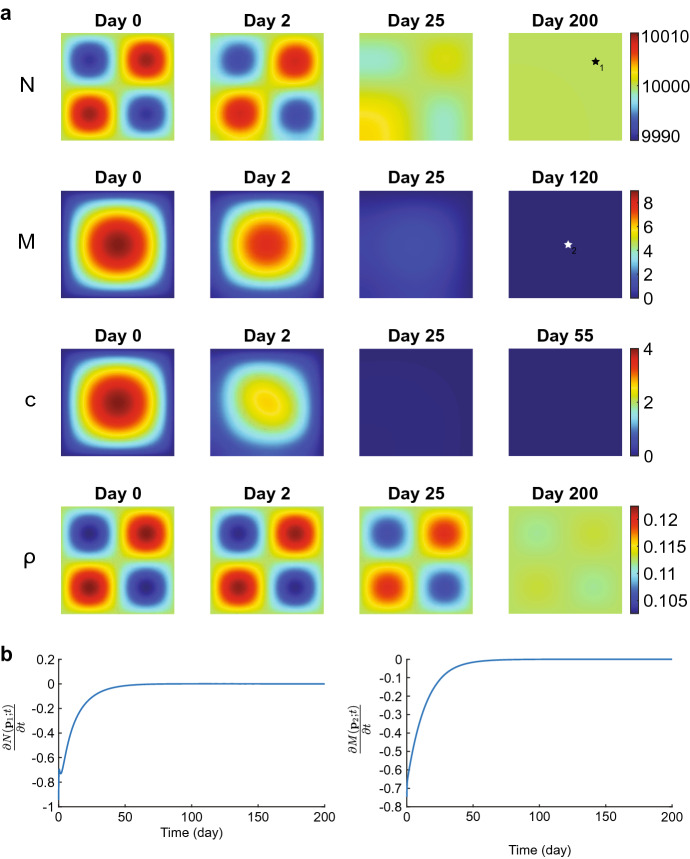
Evolution of the chemical densities for $\delta _c = 5\times 10^{-4}$ cm$^6$/(cells g day). Table 2 shows the values of the other parameters. In subfigure **a** from top to bottom, the plots show the fibroblasts (*N*), the myofibroblasts (*M*), the signaling molecules (*c*), and collagen ($\rho$). The shown domains are $(0,1)^2$ cm$^2$, and the color bars show the (myo) fibroblasts in cells/cm$^3$ and the signaling molecules and collagen in g/cm$^3$. In subfigure **b** the left and right plots show the time-dependent change of *N* at point ${\textbf{p}}_1\approx (0.75,0.75)$ and of *M* at point ${\textbf{p}}_2\approx (0.5,0.5)$, respectively (see the black and white stars in the last plots for *N* and *M* in (**a**)

In the first simulation, we take $\delta _c = 5 \times 10^{-4}$ cm$^6$/(cells g day), $k=1$ and simulate over a time interval of 200 days. We note that this parameter value meets the stability criterium. Figures 2, 3 and 4 show the results.

The displacement velocity densities move to equilibria at a rapid pace. In the first time integration, the densities drop two orders from $5\times 10^{-1}$ to $5\times 10^{-3}$ within 0.01 day ($\approx$ 15 min). The plots show that the peaks in the regions $(x,y) = \{0\le x \le 0.5 \le y\le 1 \wedge 0\le y \le 0.5 \le x\le 1\}$ merge (i.e., the top left and bottom right corners, negative values for $v_1$ and positive values for $v_2$). The peaks in the regions $(x,y)= \{0 \le x,y \le 0.5\}$ (i.e., the bottom left corners) shift to the edges of the computational domain, the one in $v_1$ shifting to the horizontal axis of symmetry, and the one in $v_2$ shifting to the vertical axis of symmetry. These variations in the displacement velocities are still visible on day 0.02. The peaks in the regions $(x,y) = \{0.5\le x,y \le 1\}$ (i.e., the top right corners) disappear within this time. The densities drop one order further towards equilibria in the next quarter of an hour. Following the stability theory, the displacement velocity densities converge to equilibria within ten days.

Figure 3 shows the evolution of the effective strains for the values of input parameters within the stability regime. The effective strain densities change from the equilibria to perturbations on the first day of the simulation because of the initial perturbations in the other variable densities. We see that diagonal tensions arise in the effective strain densities $\varepsilon _{11}({\textbf{x}},1)$ and $\varepsilon _{22}({\textbf{x}},1)$. At the same time, it is more circular for the effective strain density $\varepsilon _{12}({\textbf{x}},1)$. Here, positive and negative values alternate. These diagonal tensions disappear gradually between day ten and day 200 as the effective strain densities move to the equilibria $\varepsilon _{11}({\textbf{x}},200) = \varepsilon _{12}({\textbf{x}},200) = \varepsilon _{22}({\textbf{x}},200) = {\textbf{0}}$. Note that the theory (Thrm. 3 part 1) states that the constant state equilibria $\overline{\varepsilon _{11}},\overline{\varepsilon _{12}},\overline{\varepsilon _{22}}\in {\mathbb {R}}$ are stable.

Figure 4 shows the evolution of the chemicals for the values of input parameters within the stability regime. All the plots show that the perturbed chemical densities move gradually toward the equilibria. For the fibroblast cell density, the perturbation leaves a few fibroblasts in the origin of the computational domain on day 25. It takes up to 200 days to move the cell density toward the equilibrium $N=10^4$ cells/cm$^3$. For the myofibroblast cell density, no cells are present on day 120 as the perturbed cell density moves quickly and gradually toward the biological equilibrium $M=0$ cells/cm$^3$. The perturbed signaling molecule density moves even quicker toward the biological equilibrium $c=0$ g/cm$^3$, having the perturbations almost vanish on day 25. The perturbed collagen density takes longer to move to equilibrium as it takes more than 25 days, and on day 200, a slight perturbation is still visible. From this figure, we can conclude that the signaling molecule density first moves toward the biological equilibrium, after which the myofibroblast cell density moves toward the biological equilibrium. It takes longer for the fibroblast cell and collagen density to move toward the biological equilibria, taking more time for collagen for the current input values. Overall, the perturbations disappear gradually with stable parameter values, and the numerical method behaves stably.

From a biological perspective, minor fibroblast cell and collagen variations already initialize long-term wound healing. Fibroblasts move toward the center of the wound, and collagen regeneration takes over half a year. Variations arise in the effective strain, after which the tensions disappear.

In the next simulation, we take $\delta _c=3\times 10^{-4}$ cm$^6$/(cells g day) with $k=2$. This parameter value does not meet the stability criterium. We only present a few figures to avoid too many figures in this manuscript.

Likely, as in Fig. 2, the perturbed displacement velocity densities move gradually toward equilibria in this simulation (figure not shown). In the first step of time integration, the densities align in a similar pattern as we have seen before, decreasing from order ${\mathcal {O}}(10^{-1})$ to ${\mathcal {O}}(10^{-3})$. However, in this simulation, in the next time step, the densities decrease to order ${\mathcal {O}}(10^{-5})$ in contrast to the order ${\mathcal {O}}(10^{-4})$ in Fig. 2.

**Fig. 5:**
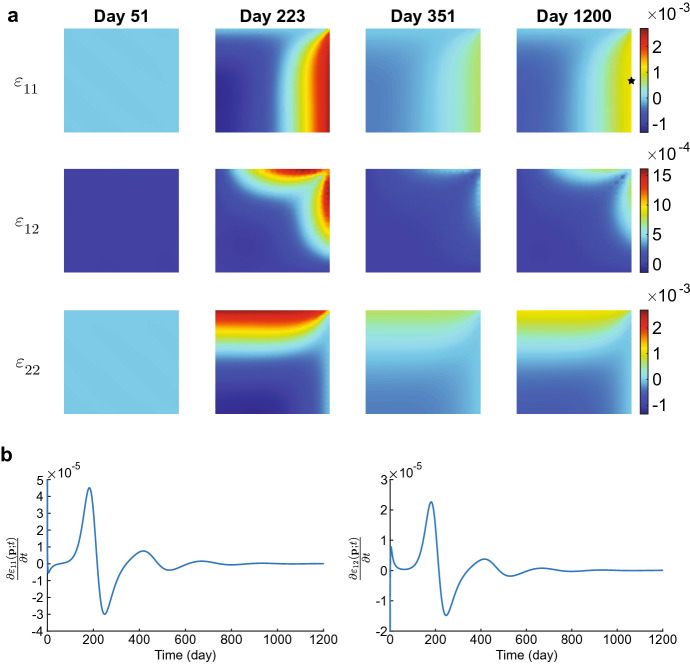
Evolution of the effective strain densities for $\delta _c = 3\times 10^{-4}$ cm$^6$/(cells g day). Table 2 shows the values of the other parameters. In subfigure **a** the upper, middle, and lower plots show the effective strains $\varepsilon _{11}, \varepsilon _{12}$, and $\varepsilon _{22}$, respectively. The shown domains are $(0,1)^2$ cm$^2$, and the color bars show the effective strains. In subfigure **b** the left and right plots show the time-dependent change of $\varepsilon _{11}$ and $\varepsilon _{12}$, respectively, at point ${\textbf{p}}\approx (1,0.5)$ (see the black star in the last plot for $\varepsilon _{11}$ in (**a**). Here, the (initial) *y*-limit is adjusted from order $10^{-3}$ to $10^{-5}$ to show the effects

We see a different evolution for the effective strains and the chemicals. We distinguish between evolution in the simulation’s early and later stages of the simulation. We see a similar pattern in the early stage of the simulation for the effective strains, as shown in Fig. 3. For the effective strains $\varepsilon _{11}$ and $\varepsilon _{22}$, the tensions in the densities are diagonal and peak on day 5. The peaks diminish in magnitude in the first 51 days (figure not shown). The effective strain $\varepsilon _{12}$ density shows the same intensity of variations on day 5, which are circular and alternating between positive and negative values, like in the stable simulation. However, Fig. 5 shows this pattern changes after 51 days.

Unlike the equilibria found in the simulation with stable parameter values, the effective strain densities increase intensely in variation up to day 223 (the second column plots). For the effective strain $\varepsilon _{11}$, this is an increase on the right edge of the computational domain; for the effective strain $\varepsilon _{22}$ on the top edge, and the effective strain $\varepsilon _{12}$ around the top right corner. In opposite directions, these densities decreased. For example, we see a decrease on the vertical axis of symmetry for the effective strain $\varepsilon _{11}$. After these peaks of intensities in the effective strain densities on day 223, the densities gradually decrease until day 351 and increase in intensity until they reach equilibrium on day 1200. Although it is difficult to see, in this 2D figure, the effective strain densities oscillate around the (new) equilibria. Compared to the simulation with stable parameter values, we see an increase in the intensity of the same order for $\varepsilon _{12}$, albeit with more significant numbers. We note that the order of magnitude may also result from the larger wavenumber ($k=2$) in the initial perturbations.

**Fig. 6:**
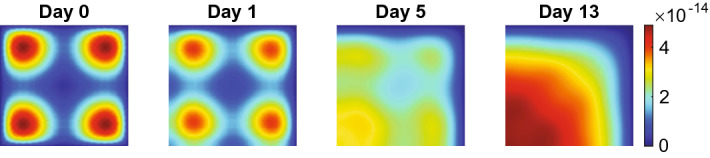
Evolution of the signaling molecule density for $\delta _c = 3\times 10^{-4}$ cm$^6$/(cells g day) in the first 13 days. Table 2 shows the values of the other parameters. The shown domains are $(0,1)^2$ cm$^2$, and the color bar shows the signaling molecules in g/cm$^3$

The early evolution of the chemicals for $\delta _c = 3\times 10^{-4}$ cm$^6$/(cells g day) is comparable to the evolution of the chemicals for $\delta _c = 5 \times 10^{-4}$ cm$^6$/(cells g day) (figure not shown). In the first 51 days, the perturbed fibroblast and myofibroblast cell densities move gradually toward the biological equilibria. The perturbed collagen density moves gradually toward the biological equilibrium in the first 119 days. However, the perturbed signaling molecule density does not reach the expected biological equilibrium. Figure 6 shows the early evolution of the signaling molecule density.

**Fig. 7:**
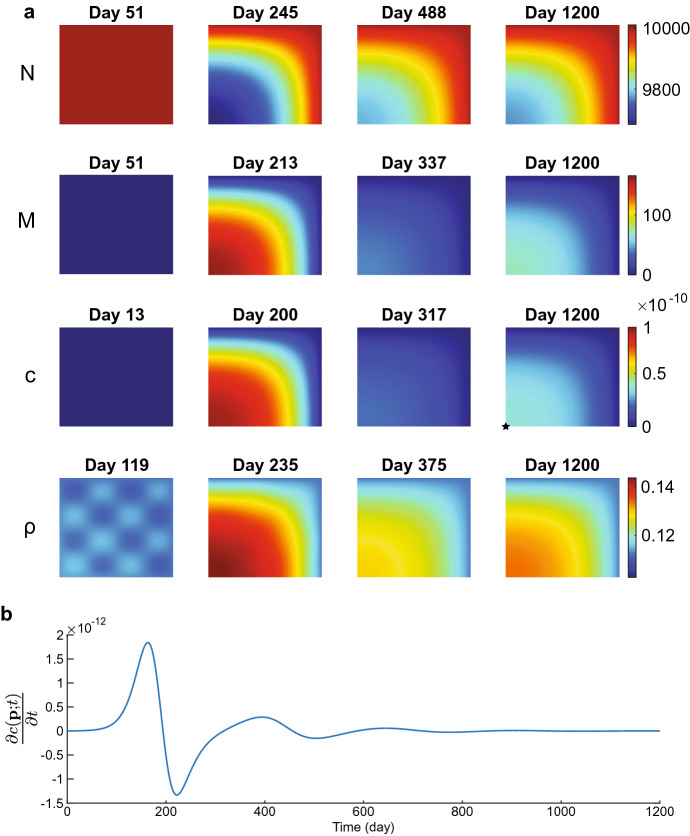
Evolution of the chemicals for $\delta _c = 3\times 10^{-4}$ cm$^6$/(cells g day). Table 2 shows the values of the other parameters. In subfigure **a** from top to bottom, the plots show the fibroblasts (*N*), the myofibroblasts (*M*), the signaling molecules (*c*), and collagen ($\rho$). The shown domains are $(0,1)^2$ cm$^2$, and the color bars show the (myo) fibroblasts in cells/cm$^3$ and the signaling molecules and collagen in g/cm$^3$. In subfigure **b** the plot shows the time-dependent change of *c* at point ${\textbf{p}}\approx (0,0)$ (see the black star in the last plot for *c* in (**a**)

Unlike the perturbed signaling molecule density evolution for stable parameter values shown in Fig. 4, the perturbations in the signaling molecule density do not disappear in the first 13 days for an unstable signaling molecule decay rate. The initial peaks of about $4\times 10^{-14}$ cm$^6$/(cells g day) decrease in the first few days. At the same time, these peaks merge and shift toward the origin as they decrease further in the first five days. The peaks continue merging, completed within 13 days; however, the signaling molecule density increases strongly in the origin of the computational domain. In the beginning, this increase does not significantly affect the other chemicals; however, after day 51, it causes a considerable difference. Figure 7 shows the evolution of the chemicals in the later stage of the simulation for $\delta _c = 3\times 10^{-4}$ cm$^6$/(cells g day).

On day 51, it seems that the fibroblast cell density is in the equilibrium $N=10^{4}$ cells/cm$^3$, the myofibroblast cell density in the equilibrium $M=0$ cells/cm$^3$, the signaling molecule density in the equilibrium $c=0$ g/cm$^3$, and the collagen density is around the equilibrium $\rho =0.1125$ g/cm$^3$. However, these densities do not stay in and around equilibria. Note the orders of the signaling molecule concentration: $10^{-10}$ g/cm$^3$ on day 200 compared to the order $10^{-14}$ g/cm$^3$ on day 13 (see Fig. 6). After day 13, in the origin of the computational domain, the signaling molecule density increases enormously until day 200, after which the density drops back toward equilibrium until day 317. The signaling molecule density then rises to a new equilibrium on day 1200, which shows a clear oscillation. Since the signaling molecule density increases so much up to day 200 in the origin of the computational domain, the fibroblast cell density decreases because of myofibroblast differentiation, and the collagen density increases there. These changes in densities are because signaling molecules stimulate the differentiation and production of myofibroblasts (Eq. (2)), stimulate the production of collagen, and inhibit the decay of collagen (Eq. (4)). Further, myofibroblasts also stimulate collagen production. The myofibroblast cell density reaches a maximum on day 213, the collagen density on day 235, and the fibroblast cell density on day 245. After the signaling molecule density reaches a minimum on day 317, we see that the myofibroblast cell density reaches a minimum on day 337, the collagen density on day 375, and the fibroblast cell density on day 448. After these days, such an oscillating effect around new equilibria is visible, which converges on day 1200. The result is a permanently reduced number of fibroblasts, a permanently increased number of myofibroblasts, and a permanently elevated concentration of signaling molecules and collagen at the origin of the computational domain (i.e., the center of the burn). Taken together, with an unstable signaling molecule decay rate not too low, the numerical method initially behaves like a stable regime. This stable behavior changes at a later stage of simulation time, where the numerical method behaves stable enough to let the chemical densities reach new equilibria in an oscillatory way.

**Fig. 8:**
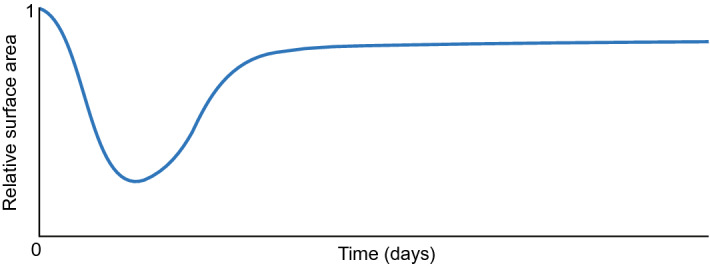
A typical schematic of the relative wound/scar area distribution

From a biological perspective, an increased expression of signaling molecules (because of their reduced decay) can lead to a period in which a wound fluctuates in contraction. This contraction fluctuation is because the number of (migrating) myofibroblasts increases and decreases. In the beginning, the wound can heal well. However, because of the continued signaling, the scar will fluctuate in thickness and stiffness because of the present collagen concentration. The scar is also highly subject to contraction because of the abundance of myofibroblasts present. The abundance of myofibroblasts and the increased collagen concentration may signify hypertrophy. Figure 8 shows an example of the contraction intensity as a function of relative wound/scar area over time.

We may associate the excessive collagen deposition with keloids, and hypertrophic scars (Tuan and Nichter 1998). Abnormal TGF-$\beta$ signaling in myofibroblasts is associated with the formation of hypertrophic scars (Zhang et al. 2020). Given our study, it is likely that such a situation arises because of a lower decay rate of signaling molecules. Furthermore, hypertrophic scars develop 1 to 2 months after injury, while keloids develop months to years after the initial injury. This period is consistent with our simulations showing that the abundance of myofibroblasts and collagen occurs after a few months, while the increased expression of signaling molecules occurs within a few weeks. Furthermore, experiments suggest that the hyper-proliferation of fibroblasts in hypertrophic scars can be reversed once their stimulation, such as the abundance of growth factors and cytokines, is abolished (Tuan and Nichter 1998). Our simulation partially reflected this. When we turn off the stimulation of signaling molecule expression at a later stage by setting the density to the biological equilibrium, we see the (myo)fibroblast cell densities and the collagen density change. The myofibroblasts seem to disappear, the collagen density recovers, and the fibroblast cell density recovers. However, with three cells/cm$^3$ myofibroblasts left in the center of the scar after 47 days after this reset, the fibroblast cell density does not increase above 9865 cells/cm$^3$, and the collagen density does not go below 0.1234 g/cm$^3$. The numerical method does not converge and decreases the time step. We, therefore, set the myofibroblast cell density to the biological equilibrium on day 1247 and see the collagen density move to equilibrium within 411 days and the fibroblast cell density within 436 days. Thus, according to this simulation, restoring the fibroblast cell and collagen density is possible when the signaling molecules and myofibroblasts disappear. Though, it then still takes over a year to repair the defects. Hence, given that the overexpression of signaling molecules occurs in the first weeks, we recommend monitoring this expression to intervene early when necessary.

In the last simulation, we take $\delta _c = 2\times 10^{-4}$ cm$^6$/(cells g day) and $k=2$. We note that this parameter value does not meet the stability criterium. While running the simulation, we see that in the first 40 days, the perturbed fibroblast cell density moves to equilibrium. After 40 days, the fibroblasts persistently differentiate into myofibroblasts, increasing the myofibroblast cell density over the whole computational domain from day 50. This differentiation happens because the signaling molecule density increases, and therefore, also the collagen density increases. Unlike in the last simulation, the displacement velocity does not vanish. For that reason, remeshing is necessary around day 80 of the simulation. Within a few days, remeshing is necessary again, and at some point, the Picard iterations do not yield convergence anymore because of continuous remeshing. Therefore, we ended this simulation. We see the same for $k=1$.

From a biological point of view, the human body protects against lowering the signaling molecule decay rate to this extent to prevent such a non-realistic occurrence where collagen will cause the tissue to rupture because of excessive production.

To conclude this section, the two-dimensional morphoelastic model for skin contraction is stable under the condition that the signaling molecule decay rate is not reduced too far below the limit $\delta _c \ge \frac{k_c}{a_c^{II} {\overline{\rho }}}$.

## Discussion and conclusion

For the discussion, we focus on adapting the model to bring it closer to reality and on applying neural networks to bring the model into practice.

Since hypertrophy is one common complication after burn injuries, it would be interesting to incorporate hypertrophy into this morphoelastic model. Koppenol made some progress in incorporating hypertrophy (Koppenol et al. 2017), though this model considers a compressible neo-Hookean solid instead of a morphoelastic solid. Therefore, these models need to be merged so that a three-dimensional morphoelastic model simulates contraction and hypertrophy. In addition, hypertrophy depends highly on angiogenesis, which can also be considered. References (Valero et al. 2012; Vermolen and Javierre 2011) provide a good start for this.

In a three-dimensional setting, computation times increase rapidly. In order to decrease computation, the computational domain could be reduced by only incorporating the wound or scar region. If so, we need to adjust the boundary conditions and compare the results to the results of the whole computation domain where the boundaries are ‘sufficiently far away’ from the wound boundaries. We could model the wound boundaries as elastic springs. Furthermore, rotational symmetry and isogeometric analysis offer solutions to the curse of dimensionality and the decreasing quality of the moving mesh.

Further, post-burn contraction and the persistence of myofibroblasts in hypertrophic scars depend on stretching, pulling, and tissue stiffness. If we incorporate these features into the model, we can study pulling and stretching forces because of children’s growth and as a consequence of the joints’ daily (repetitive) motion. We can incorporate these forces in the body force in Eq. (5) and in the boundary conditions.

As far as we know, it is uncertain if myofibroblasts proliferate. At least, we know myofibroblasts proliferate at a lower rate than fibroblasts. There is a difference between proto-myofibroblasts and fully differentiated myofibroblasts. One might discuss that proto-myofibroblasts either proliferate or contract the tissue and that fully differentiated myofibroblasts only contract the tissue. Although it may not have been demonstrated that fully differentiated myofibroblasts do not proliferate, we could nevertheless assume that these cells only contract if we, for example, add an equation for the proto-myofibroblasts that do proliferate. We can also easily consider that myofibroblasts, in response to TGF-$\beta$, move more slowly than fibroblasts do (Thampatty and Wang 2006) and that in vitro myofibroblasts can differentiate back to fibroblasts under the influence of Prostaglandin E2 (Garrison et al. 2013). Myofibroblast de-differentiation has not been observed in vivo; therefore, this has not been accounted for in the current model formulation. The impact of the inclusion of this feature needs to be quantified.

**Table 2:** Overview of the parameters used for the simulations. Shown are the symbols, values, dimensions, and references

Symbol	Value	Dimension	References
$D_c$	$2.88\times 10^{-3}$	cm$^2$/day	Haugh (2006)
$D_F$	$10^{-7}$	cm$^5$/(cells day)	Sillman et al. (2003)
$\chi _F$	$2\times 10^{-3}$	cm$^5$/(g day)	Murphy et al. (2012)
$k_c$	$4\times 10^{-13}$	g/(cells day)	Olsen et al. (1995)
$r_F$	$9.24\times 10^{-1}$	cm$^{3q}$/(cells$^q$ day)	Alberts et al. (1989) and Gosh et al. (2007)
$r_F^{\text {max}}$	2	–	Strutz et al. (2001)
$k_\rho$	$7.6\times 10^{-8}$	g/(cells day)	[NC]
$k_\rho ^{\text {max}}$	10	–	Olsen et al. (1995)
$a_c^{I}$	$10^{-8}$	g/cm$^3$	Olsen et al. (1995)
$a_c^{II}$	$10^{-8}$	cm$^3$/g	Olsen et al. (1995)
$a_c^{III}$	$2\times 10^8$	g/cm$^3$	Overall et al. (1991)
$a_c^{IV}$	$10^{-9}$	g/cm$^3$	Roberts et al. (1986)
$\eta ^I$	2	–	Rudolph and Vande Berg (1991)
$\eta ^{II}$	$5\times 10^{-1}$	–	Koppenol and Vermolen (2017)
$k_F$	$1.08\times 10^{7}$	cm$^3$/(g day)	Desmoulière et al. (1993)
$\kappa _F$	$10^{-6}$	cm$^3$/cells	Vande Berg et al. (1989)
*q*	$-4.151\times 10^{-1}$	–	[NC]
$\delta _c$	$5\times 10^{-4}$	cm$^6$/(cells g day)	Olsen et al. (1995)
$\delta _N$	$2\times 10^{-2}$	/day	Olsen et al. (1995)
$\delta _M$	$6\times 10^{-2}$	/day	Koppenol et al. (2017)
$\delta _\rho$	$6\times 10^{-6}$	cm$^6$/(cells g day)	Koppenol et al. (2017)
${\overline{N}}$	$10^{4}$	cells/cm$^3$	Olsen et al. (1995)
${\overline{M}}$	0	cells/cm$^3$	Olsen et al. (1995)
${\overline{c}}$	0	g/cm$^3$	Koppenol et al. (2017)
${\overline{\rho }}$	$1.125\times 10^{-1}$	g/cm$^3$	Olsen et al. (1995)
$\rho _t$	1.09	g/cm$^3$	Wrobel et al. (2009)
$\mu _1$	$10^2$	(N day)/cm$^2$	Koppenol and Vermolen (2017)
$\mu _2$	$10^2$	(N day)/cm$^2$	Koppenol and Vermolen (2017)
$\nu$	$4.9\times 10^{-1}$	–	Liang and Boppart (2010)
*E*	$$3.2\times 10$$	N/((g cm)$$^{0.5})$$	Liang and Boppart (2010)
$\xi$	$4.4\times 10^{-2}$	(N g)/(cells cm$^2$)	Maskarinec et al. (2009) and Wrobel et al. (2002)
*R*	$9.95\times 10^{-1}$	g/cm$^3$	Koppenol and Vermolen (2017)
$\zeta$	$4\times 10^2$	cm$^6$/(cells g day)	Koppenol and Vermolen (2017)

Here NC denotes that the value of the parameter is a consequence because of the chosen values for other parameters

The state change between fibroblasts and myofibroblasts is more gradual than has been modeled here. The intermediate state of proto-myofibroblasts could also be implemented into the modeling framework. If one seeks to incorporate this intermediate state, then it is important to know the behavior of proto-myofibroblasts. This model extension also needs additional input parameters, which could be prone to uncertainty. The current model allows the modification of the constitutive law between stress and strain. One could revise the elasticity part, which is currently modeled as linear (Hookean) and hyperelastic, such that large strains are allowed from a physical perspective. This extension adapts Eq. (7) (Hall 2008; Koppenol 2017), adding more nonlinearity to the model and making the stability analysis even more complicated.

Furthermore, this nonlinearity will also add some complexity to the numerical algorithms. Another extension concerns the implementation of different collagen types. This addition could make sense since myofibroblasts produce collagen type III in large quantities, whereas fibroblasts produce collagen I (embryonic). Collagen III is known to be aligned anisotropically (directed), whereas collagen I possess a more isotropic (random) alignment. This difference in alignment causes these collagen types to exhibit significantly different mechanical properties and behavior. In order to implement the distinction between the two types of collagen, more quantitative information should be available regarding the production and mechanical properties of these collagen types. In general, model extensions such that the underlying biology and physics are described in more detail lead to the need for additional input parameters, which may have yet to be measured or even be hard to measure. In addition, these extensions often lead to additional uncertainty; hence, such extensions only sometimes improve the quality of the model. However, adding the distinction between the two collagen types will lead to a better description of the development of the contraction, particularly the development of hypertrophic scars.

Given that the finite element simulations take much time and many Monte Carlo-based predictions are needed, for example, to reach personalized healthcare, we prefer the application of neural networks. We can train a neural network on the data we generate with the finite element method and then make predictions about contraction. Training a neural network takes time; however, it gives predictions at ultra-high computational speed once trained. This quick speed will make running an application on a smartphone or tablet possible.

There is much variation between burns and patients, which entails uncertainty. These uncertainties are twofold. On the one hand, some parameters are not or hardly measurable. For this purpose, we must apply inverse modeling to estimate parameter values and link the results to (clinical) experiments. On the other hand, parameter values are patient-dependent, so we need to perform uncertainty quantification. Therefore, inter-parameter dependency and patient-specific factors need to be investigated.

For the sake of parameter sensitivity analysis, we need to perform Monte Carlo simulations, and for higher-dimensional frameworks, this can become a challenge because of computational complexity. Hence, we require implementing the finite element method in a high-level programming language like C++. We can also consider clever Monte Carlo techniques based on many simulations with low numerical resolution and a few with high numerical resolution. For these Monte Carlo simulations, it is essential to consider both the model’s sensitivity and stability. However, we need high-quality finite element simulations to train a neural network. Therefore, for Monte Carlo simulations only, we can use the method of many low numerical resolution simulations and a few simulations with high numerical resolution. Training a neural network in higher-dimensional frameworks requires only high numerical-resolution simulations.

Besides training a neural network on the generated data from simulations, it is also an option to train directly on clinical data. We can think of datasets that provide burn images as input, supplemented with the patient’s data, the burn’s location and cause, and the course of contraction, contracture, and hypertrophy. Creating such datasets requires a systematic follow-up or a hybrid approach that works with variable data when the data is limited.

For such a neural network, we can fit the wound using a convolutional neural network that takes images of the initial wound. For these images’ contours and other features, we can use pixel-based methods, shape similarity (Andreou and Sgouros 2005), and shape matching (Veltkamp 2001).

This study presented a stability analysis for the fully continuous and semi-discrete version of the two-dimensional model for post-burn contraction. We could analytically determine the eigenvalues, which is possible because the linearised equations (13)–(15) leave out other variables after accounting for the equilibria values. As a result, some eigenvalues meet the stability constraints independent of the chosen value for the parameters, given that the parameters involved are positive and realistic. We have illustrated that for the parameter range that we are interested in, there is a region around the origin of the $(\overline{\varepsilon _{11}}, \overline{\varepsilon _{22}}, \overline{\varepsilon _{12}})$–space where the model is stable with respect to perturbations with all wave frequencies. This condition is sufficient, meaning the current analysis does not exclude any other stable equilibria. Nonlinear effects will, at most, be able to induce constant-state instabilities for the effective strain. Further, an important stability constraint states that the model is stable for low signaling molecule decay rates, though not too low.

We have shown consistency between the semi-discrete model’s eigenvalues and the continuous model’s eigenvalues. If the equilibrium solution to the continuous problem is stable, then the equilibrium to the semi-discrete problem is stable under the present discretization. The obtained eigenvalues of the system establish the convergence rate towards the equilibrium. We have assessed the convergence of the numerical method experimentally, in which the order of convergence is above ${\mathcal {O}}(h^2)$. Since the difference between the chemical eigenvalues from the continuous and semi-discrete problem is of the order ${\mathcal {O}}(h^2)$, the convergence rates towards the equilibrium differ by an order ${\mathcal {O}}(h^2)$. This result is better than expected since the discretization method should have local truncation errors of order ${\mathcal {O}}(h^2)$.

Using numerical simulations, we validated the stability constraints derived from the analysis. If the input values satisfy the stability criterion, the model behaves stable given these stable parameter values.

The model can numerically be unstable if the parameters do not meet the signaling molecule stability constraint. If $\delta _c < k_c/(a_c^{II}{\overline{\rho }})$ is not too far below the bound, then, initially, the model seems stable and the healing proper. The displacement velocity perturbations vanish quickly, and the signaling molecule perturbation shifts such that the density peak moves to the center of the wound in the first 13 days. One would expect this density to decrease from that day on, given that the other chemicals seem to reach equilibria. However, shortly after 13 days, the signaling molecule density moves away from equilibrium, affecting all the variables except displacement velocity. The distributions and densities move away from the expected equilibria and oscillate around new equilibria, where the densities remain. We have linked this situation to real-life occurrences of hypertrophic scars and keloids. By reverting the stimulation of matrix production and myofibroblast differentiation by setting the signaling molecule density to (healthy) equilibrium and removing the myofibroblasts, we have provided experimental evidence, from a mathematical point of view, that one can indeed restore the fibroblast cell and collagen density to healthy equilibria.

If the signaling molecule decay rate is too far below the stability limit, then the numerical method does not converge and loops over Picard iterations while remeshing. Taken together, the numerical model fully reproduces the stability constraints.

### Appendix: The derivation of equation (49)

Substitution of variations from Eq. (47) into Eq. (45) yields

59 $$\begin{aligned} \begin{aligned} \rho _t\lambda _{v_1}&= \sum _{\beta =1}^{N_x}\sum _{\gamma =1}^{N_y}{\hat{v}}^1_{\beta ,\gamma }\left[ - \left( \mu _1+\mu _2\right) \frac{e^{-2\pi \beta (k-1)hi}e^{-2\pi \gamma jhi}-2e^{-2\pi \beta khi}e^{-2\pi \gamma jhi}+e^{-2\pi \beta (k+1)hi}e^{-2\pi \gamma jhi}}{h^2}\, \right. \\&\quad -\left. \frac{\mu _1}{2}\frac{e^{-2\pi \beta khi}e^{-2\pi \gamma (j-1)hi} - 2e^{-2\pi \beta khi}e^{-2\pi \gamma jhi} + e^{-2\pi \beta khi}e^{-2\pi \gamma (j+1)hi}}{h^2} \right] \\&\quad -\left[ \frac{\mu _1}{2}+\mu _2\right] \sum _{\beta =1}^{N_x}\sum _{\gamma =1}^{N_y}{\hat{v}}^2_{\beta ,\gamma }\left[ \frac{e^{-2\pi \beta (k-1)hi}e^{-2\pi \gamma (j-1)hi} - e^{-2\pi \beta (k-1)hi}e^{-2\pi \gamma (j+1)hi}}{4h^2}\, \right. \\&\quad +\dots \left. \frac{-e^{-2\pi \beta (k+1)hi}e^{-2\pi \gamma (j-1)hi} + e^{-2\pi \beta (k+1)hi}e^{-2\pi \gamma (j+1)hi}}{4h^2}\right] \\&\quad -\frac{E\sqrt{{\overline{\rho }}}}{1+\nu }\sum _{\beta =1}^{N_x}\sum _{\gamma =1}^{N_y}\left[ {\hat{\varepsilon }}^{12}_{\beta ,\gamma }\frac{e^{-2\pi \beta khi}e^{-2\pi \gamma (j+1)hi} - e^{-2\pi \beta khi}e^{-2\pi \gamma (j-1)hi}}{2h} \,\right. \\&\quad +\left. \frac{1-\nu }{1-2\nu }{\hat{\varepsilon }}^{11}_\beta \frac{e^{-2\pi \beta (k+1)hi}e^{-2\pi \gamma jhi} - e^{-2\pi \beta (k-1)hi}e^{-2\pi \gamma jhi}}{2h} \,\right. \\&\quad +\left. \frac{\nu }{1-2\nu }{\hat{\varepsilon }}^{22}_\beta \frac{e^{-2\pi \beta (k+1)hi}e^{-2\pi \gamma jhi} - e^{-2\pi \beta (k-1)hi}e^{-2\pi \gamma jhi}}{2h} \right] \\&\quad -\frac{E}{2\sqrt{{\overline{\rho }}}(1+\nu )}\left[ \overline{\varepsilon _{12}} + \overline{\varepsilon _{11}} + \frac{\nu }{1-2\nu }\left( \overline{\varepsilon _{11}} + \overline{\varepsilon _{22}}\right) \right] \sum _{\beta =1}^{N_x}\sum _{\gamma =1}^{N_y}{\hat{\rho }}_{\beta ,\gamma }\frac{e^{-2\pi \beta (k+1)hi}e^{-2\pi \gamma jhi} - e^{-2\pi \beta (k-1)hi}e^{-2\pi \gamma jhi}}{2h} \\&\quad -\xi \frac{{\overline{\rho }}}{R^2+{\overline{\rho }}^2}\sum _{\beta =1}^{N_x}\sum _{\gamma =1}^{N_y}{\hat{M}}_{\beta ,\gamma }\frac{e^{-2\pi \beta (k+1)hi}e^{-2\pi \gamma jhi} - e^{-2\pi \beta (k-1)hi}e^{-2\pi \gamma jhi}}{2h}. \end{aligned}\nonumber \\ \end{aligned}$$

Multiplication by $e^{2\pi lkhi}e^{2\pi pjhi}$ and double orthonormalization yields

60 $$\begin{aligned} \begin{aligned} \rho _t\lambda v^1_{i,j}&= \left[ - \left( \mu _1+\mu _2\right) \frac{e^{2\pi l hi} -2 + e^{-2\pi l hi}}{h^2} - \frac{\mu _1}{2}\frac{e^{2\pi phi} - 2 + e^{-2\pi phi}}{h^2} \right] {\hat{v}}^1_{\varvec{\beta }} \, \\&\quad -\left[ \frac{\mu _1}{2}+\mu _2\right] \left[ \frac{e^{2\pi lhi}e^{2\pi phi} - e^{2\pi lhi}e^{-2\pi phi}}{4h^2}\,\right. \\&\quad +\left. \frac{-e^{-2\pi lhi}e^{2\pi phi} + e^{-2\pi lhi}e^{-2\pi phi}}{4h^2}\right] {\hat{v}}^2_{\varvec{\beta }} \,\\&\quad -\frac{E\sqrt{{\overline{\rho }}}}{1+\nu }\left[ {\hat{\varepsilon }}^{12}_{\varvec{\beta }}\frac{e^{-2\pi phi} - e^{2\pi phi}}{2h} + \frac{1-\nu }{1-2\nu }{\hat{\varepsilon }}^{11}_{\varvec{\beta }}\frac{e^{-2\pi lhi} - e^{2\pi phi}}{2h} \,\right. \\&\quad +\left. \frac{\nu }{1-2\nu }{\hat{\varepsilon }}^{22}_{\varvec{\beta }}\frac{e^{-2\pi lhi} - e^{2\pi lhi}}{2h} \right] \\&\quad -\frac{E}{2\sqrt{{\overline{\rho }}}(1+\nu )}\left[ \overline{\varepsilon _{12}} + \overline{\varepsilon _{11}} + \frac{\nu }{1-2\nu }\left( \overline{\varepsilon _{11}} + \overline{\varepsilon _{22}}\right) \right] \frac{e^{-2\pi lhi} - e^{2\pi lhi}}{2h}{\hat{\rho }}_{\varvec{\beta }} \, \\&\quad -\xi \frac{{\overline{\rho }}}{R^2+{\overline{\rho }}^2}\frac{e^{-2\pi lhi} - e^{2\pi lhi}}{2h}{\hat{M}}_{\varvec{\beta }}, \end{aligned}\nonumber \\ \end{aligned}$$

for the displacement velocity $v_1$. Application of Euler’s formula, and using $2-2\cos (2\pi lx)=4\sin ^2(\pi lx)$ and $\left[ e^{ix}e^{iy}-e^{ix}e^{-iy}-e^{-ix}e^{iy}+e^{-ix}e^{-iy}\right] /4 = -\sin (x)\sin (y)$ yields equation (49).

## Data Availability

All relevant data will be available in the 4TU.Centre for Research Data.

## Footnotes

1 On a molecular basis, this is the contraction mechanism which is the interaction between the actin and myosin filaments that generates their movement relative to one another (Cooper 2000).

## Code Availability

All relevant code will be available in the 4TU.Centre for Research Data.

## References

[CR1] Alberts B, Bray D, Lewis J, Raff M, Roberts K, Watson J (1989). The molecular biology of the cell.

[CR2] Andreou I, Sgouros N (2005). Computing, explaining and visualizing shape similarity in content-based image retrieval. Inf Process Manag.

[CR3] Barocas V, Tranquillo R (1997). An anisotropic biphasic theory of tissue-equivalent mechanics: the interplay among cell traction, fibrillar network deformation, fibril alignment, and cell contact guidance. J Biomech Eng.

[CR4] Caroli B, Caroli C, Roulet B (1986). The Mullins–Sekerka instability in directional solidification of thin samples. J Cryst Growth.

[CR5] Cooper G (2000). The cell: a molecular approach.

[CR6] Dallon J, Sherrat J, Maini P (1999). Mathematical modelling of extracellular matrix dynamics using discrete cells: fiber orientation and tissue regeneration. J Theor Biol.

[CR7] Desmoulière A, Geinoz A, Gabbiani F, Gabbiani G (1993). Transforming growth factor-beta 1 induces alpha-smooth muscle actin expression in granulation tissue myofibroblasts and in quiescent and growing cultured fibroblasts. J Cell Biol.

[CR8] Desmoulière A, Redard M, Darby I, Gabbiani G (1995). Apoptosis mediates the decrease in cellularity during the transition between granulation tissue and scar. Am J Pathol.

[CR9] Dziuk G, Elliot C (2007). Finite elements on evolving surfaces. IMA J Numer Anal.

[CR10] Egberts G, Smits D, Vermolen F, Zuijlen Pv (2021a) Some mathematical properties of morphoelasticity. In: Vermolen FJ, Vuik C (eds) Numerical mathematics and advanced applications ENUMATH 2019. Lecture notes in computational science and engineering, vol 139. Springer, Cham. 10.1007/978-3-030-55874-1_111

[CR11] Egberts G, Vermolen F, van Zuijlen P (2021b) Stability of a one-dimensional morphoelastic model for post-burn contraction. J Math Biol 83(3):24. 10.1007/s00285-021-01648-5 10.1007/s00285-021-01648-5 PMC8342404 34355270

[CR12] Garrison G, Huang S, Okunishi K, Scott J, Penke L, Scruggs A, Golden M (2013). Reversal of myofibroblast differentiation by prostaglandin E2. Am J Respir Cell Mol Biol.

[CR13] Ghosh K, Pan Z, Guan E, Ge S, Liu Y, Nakamura T, Ren XD, Rafailovich M, Clark RA (2007). Cell adaptation to a physiologically relevant ECM mimic with different viscoelastic properties. Biomaterials.

[CR14] Hall C (2008) Modelling of some biological materials using continuum mechanics. Ph.D. thesis, Queensland University of Technology

[CR15] Haugh J (2006). Deterministic model of dermal wound invasion incorporating receptor-mediated signal transduction and spatial gradient sensing. Biophys J.

[CR16] Hillen T, Painter K (2008). A user’s guide to PDE models for chemotaxis. J Math Biol.

[CR17] Koppenol D (2017) Biomedical implications from mathematical models for the simulation of dermal wound healing. Ph.D. thesis, Delft University of Technology

[CR18] Koppenol D, Vermolen F (2017). Biomedical implications from a morphoelastic continuum model for the simulation of contracture formation in skin grafts that cover excised burns. Biomech Model Mechanobiol.

[CR19] Koppenol D, Vermolen F, Niessen F (2017). A mathematical model for the simulation of the formation and the subsequent regression of hypertrophic scar tissue after dermal wounding. Biomech Model Mechanobiol.

[CR20] Lang T, Zhao R, Kim A, Wijewardena A, Vandervord J, Xue M, Jackson C (2019) A critical update of the assessment and acute management of patients with severe burns. Adv Wound Care 8(12):607–633. 10.1089/wound.2019.0963 10.1089/wound.2019.0963 PMC6904939 31827977

[CR21] Liang X, Boppart S (2010). Biomechanical properties of in vivo human skin from dynamic optical coherence elastography. IEEE Trans Biomed Eng.

[CR22] Maskarinec S, Franck C, Tirell D, Ravichandran G (2009). Quantifying cellular traction forces in three dimensions. Proc Natl Acad Sci.

[CR23] McDougall S, Dallon J, Sherrat J, Maini P (2006). Fibroblast migration and collagen deposition during dermal wound healing: mathematical modelling and clinical implications. Philos Trans R Soc A Math Phys Eng Sci.

[CR24] Menon S, Hall C, McCue S, McElwain D (2017). A model for one-dimensional morphoelasticity and its application to fibroblast-populated collagen lattices. Biomech Model Mechanobiol.

[CR25] Murphy K, Hall C, Maini P, McCue S, MacElwain D (2012). A fibrocontractive mechanochemical model of dermal wound closure incorporating realistic growth factor kinetics. Bull Math Biol.

[CR26] Olsen L, Sherratt J, Maini P (1995). A mechanochemical model for adult dermal wound contraction and the permanence of the contracted tissue displacement profile. J Theor Biol.

[CR27] Overall C, Wrana J, Sodek J (1991). Transcriptional and post-transcriptional regulation of 72-kda gelatinase/ type iv collagenase by transforming growth factor-beta in human fibroblasts. J Biol Chem.

[CR28] Postlethwaite A, Keski-Oja J, Moses H, Kang A (1987). Stimulation of the chemotactic migration of human fibroblasts by transforming growth factor beta. J Exp Med.

[CR29] Ramtani S (2004). Mechanical modelling of cell/ECM and cell/cell interactions during the contraction of a fibroblast-populated collagen microsphere: theory and model simulation. J Biomech.

[CR30] Roberts A, Sporn M, Assoian R, Smith J, Roche N, Wakefield L, Heine U, Liotta L, Falanga V, Kehrl J (1986). Transforming growth factor type beta: rapid induction of fibrosis and angiogenesis in vivo and stimulation of collagen formation in vitro. Proc Natl Acad Sci.

[CR31] Rudolph R, Vande Berg J (1991). The myofibroblast in Dupuytren’s contracture. J. Hand Clin.

[CR32] Sillman A, Quang D, Farboud B, Fang K, Nuccitelli R, Isseroff R (2003). Human dermal fibroblasts do not exhibit directional migration on collagen I in direct-current electric fields of physiological strength. Exp Dermatol.

[CR33] Stéphanou A, Volpert V (2015). Hybrid modelling in biology: a classification review. Math Model Nat Phenom.

[CR34] Strutz F, Zeisberg M, Renziehausen A, Raschke B, Becker V, Van Kooten C, Müller G (2001). TGF-$$\beta $$1 induces proliferation in human renal fibroblasts via induction of basic fibroblast growth factor (FGF-2). Kidney International.

[CR35] Thampatty B, Wang J (2006). A new approach to study fibroblast migration. Cell Motil Cytoskelet.

[CR36] Tomasek J, Gabbiani G, Hinz B, Chaponnier C, Brown R (2002). Myofibroblasts and mechano-regulation of connective tissue remodelling. Nat Rev Mol Cell Biol.

[CR37] Tranquillo R, Murray J (1992). Continuum model of fibroblast-driven wound contraction: inflammation-mediation. J Theor Biol.

[CR38] Tuan T, Nichter L (1998). The molecular basis of keloid and hypertrophic scar formation. Mol Med Today.

[CR39] Valero C, Javierre E, García-Aznar JM, Gómez-Benito MJ (2012). Numerical modelling of the angiogenesis process in wound contraction. Biomech Model Mechanobiol.

[CR40] Van Kan J, Segal A, Vermolen F (2014). Numerical methods in scientific computing.

[CR41] Vande Berg J, Rudolph R, Poolman W, Disharoon D (1989). Comparative growth dynamics and actin concentration between cultured human myofibroblasts from granulating wounds and dermal fibroblasts from normal skin. Lab Invest.

[CR42] Veltkamp R (2001) Shape matching: similarity measures and algorithms. In: Proceedings international conference on shape modeling and applications. IEEE Computer Society. 10.1109/sma.2001.923389

[CR43] Vermolen F, Javierre E (2011). A finite-element model for healing of cutaneous wounds combining contraction, angiogenesis and closure. J Math Biol.

[CR44] Wang Y, Beekman J, Hew J, Jackson S, Issler-Fisher A, Parungao R, Lajevardi S, Li Z, Maitz P (2018). Burn injury: challenges and advances in burn wound healing, infection, pain and scarring. Adv Drug Del Rev.

[CR45] Wrobel L, Fray T, Molloy J, Adams J, Armitage M, Sparrow J (2002). Contractility of single human dermal myofibroblasts and fibroblasts. Cell Motil Cytoskelet.

[CR46] Wrobel L *et al* (2009) Anex A: Table A.1. Annals of the ICRP Publication 110, vol 39, no 2, pp 48–51

[CR47] Zhang T, Wang X, Wang Z, Lou D, Fang Q, Hu Y, Zhao W, Zhang L, Wu L, Tan W (2020). Current potential therapeutic strategies targeting the TGF-$$\beta $$/Smad signaling pathway to attenuate keloid and hypertrophic scar formation. Biomed Pharmacother.

